# Therapeutic potential of oncolytic viruses in hematopoietic malignancies: from preclinical insights to clinical realities

**DOI:** 10.3389/fmicb.2026.1925346

**Published:** 2026-09-29

**Authors:** Sebastian Erkens, Dawit Belai Ghebrehiwot, Julius Knierer, Xiaofang Wang, Anja Ehrhardt, Wenli Zhang

**Affiliations:** 1 Department of Human Medicine, Center for Biomedical Education and Research (ZBAF), Witten/Herdecke University, Witten, Germany; 2 Department of Hematology, Tianjin Medical University Cancer Institute and Hospital, Tianjin, China

**Keywords:** clinical trial, combination therapy, diffuse large B-cell lymphoma, hematopoietic malignancies, immuno-virotherapy, oncolytic viruses, systemic administration

## Abstract

While certain forms of lymphoma—such as classical Hodgkin lymphoma—are classified as curable, with over half of all patients achieving remission following first-line therapy, a significant proportion of patients still suffer from relapsed or refractory disease. Through autologous stem cell transplantation (ASCT) and chimeric antigen receptor (CAR) T-cell therapy can significantly improve survival rates, their widespread clinical utility is constrained by logistical bottlenecks, manufacturing complexities, and high financial burdens. Similarly, antibody-drug conjugates and bispecific antibodies have achieved striking success against hematologic malignancies, but their clinical adoption is often hindered by the requirement for continuous infusion. Consequently, systemically delivered oncolytic viruses engineered to express immunotherapeutic elements have emerged as highly innovative alternatives. This review provides a comprehensive overview of the current landscape and outlines the therapeutic potential of oncolytic virotherapy in the fight against hematopoietic malignancies. We cross-examined a robust dataset of over 100 preclinical investigations to critically assess the therapeutic efficacy and safety profiles of more than ten diverse oncolytic virus platforms developed for blood cancer treatment, alongside multiple early-phase clinical trials that are completed or currently underway. By synthesizing these preclinical and clinical data, we propose three critical development pathways—enhancing target specificity, arming viral vectors, and designing synergistic combination regimens—to accelerate the translation of clinically viable oncolytic viruses for hematologic oncology.

## Introduction and methodology

Oncolytic viruses (OVs) have been widely studied in solid tumors, typically through intratumoral administration. In contrast, hematopoietic malignancies—which inherently require systemic drug delivery rather than local injection—have historically received less attention as primary targets for OV therapy. In this review, we explored data from clinical applications and trials, tracing evidence back through preclinical and basic research. By analyzing the current landscape of utilizing OVs to treat hematopoietic malignancies, we highlight this systemic targeting paradigm as possessing a uniquely favorable therapeutic window for advanced OV research.

This mini-review was conducted by searching the PubMed and ClinicalTrials.gov databases up to July 2026 using the terms “oncolytic virus” and “hematopoietic malignancies” or “blood cancer” or “lymphoma” or “leukemia” or “multiple myeloma.” Additional references were identified through citation tracking of relevant reviews and primary studies, prioritizing the most recent and clinically significant trials, preclinical advances, and mechanistic insights.

Inclusion criteria: Studies were included if they evaluated live oncolytic virus platforms specifically targeting hematopoietic malignancies, lymphomas, leukemias, or multiple myeloma in preclinical models or clinical trials. Only articles published in English were considered, with no restrictions applied to publication date or study type.

Exclusion criteria: Non-English articles, abstract-only publications lacking primary data, and studies focusing strictly on solid tumors without hematological evaluations were excluded.

Initial database searches yielded a combined pool of records. Duplicates were removed, and the remaining articles were screened by the first reviewer based on title and abstract. Full texts of potentially eligible studies were then independently assessed against the inclusion criteria by a second reviewer. Any discrepancies regarding article selection or data extraction were resolved through consensus discussion between both reviewers prior to final inclusion Supplementary Tables and Figure 1. For each eligible study, information regarding study design, disease indication, efficacy-related outcomes, safety profiles, and adverse events associated with oncolytic virotherapy was collected and summarized.

**Figure 1:**
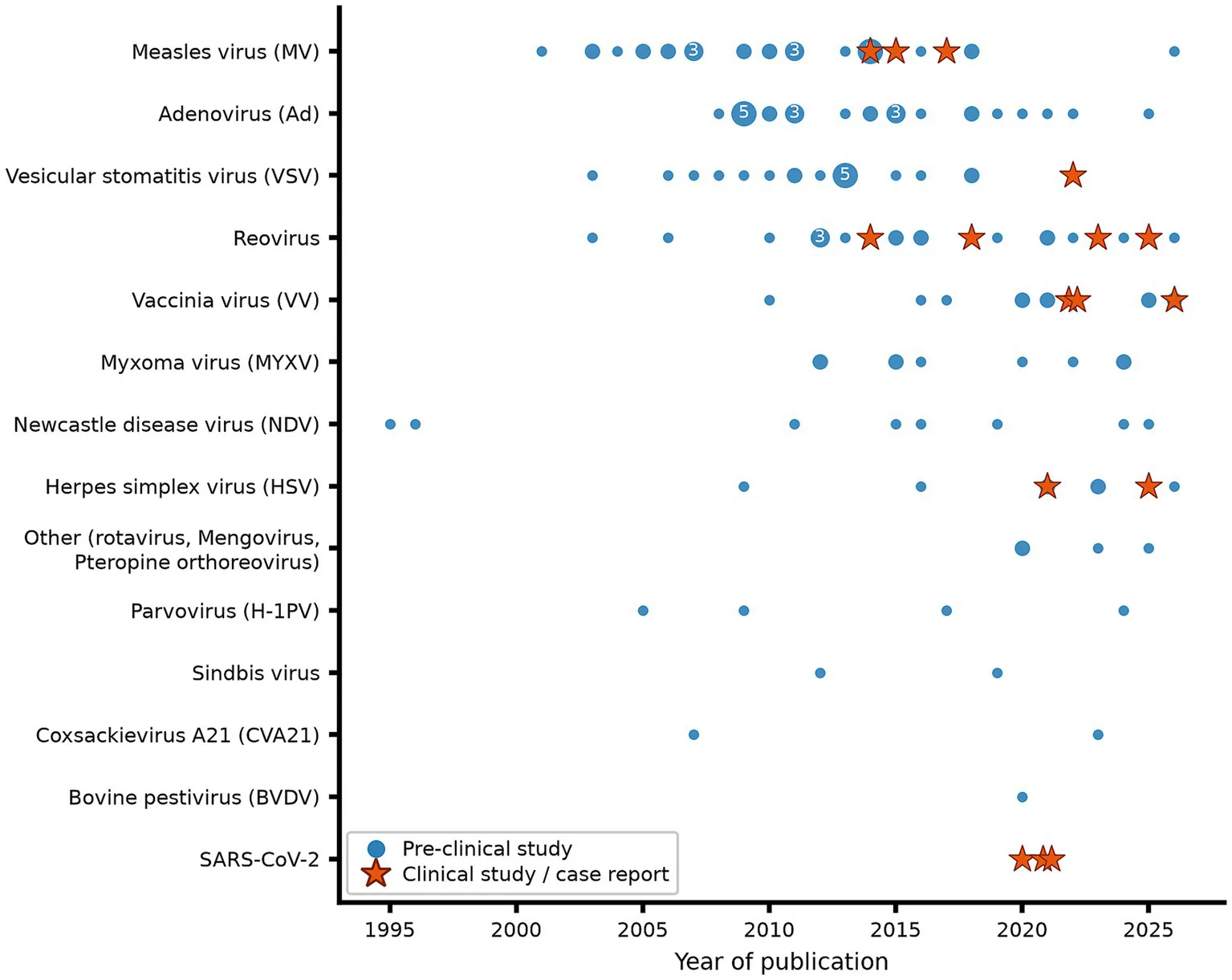
Pre-clinical and clinical studies using OVs to treat blood cancers. Pre-clinical and clinical studies using OVs to treat blood cancers recorded in the PubMed and ClinicalTrials.gov databases in the past three decades are distributed according to the publication year (X-axis) and virus types (Y-axis). For preclinical studies, the numbers inside the blue circles indicate the number of studies conducted for each OV in that year; those without numbers indicate that only one study was conducted. The Pteropine orthoreovirus (Sikamat virus) grouped under ‘Other’ is a distinct bat-borne species and not the mammalian reovirus (pelareorep) shown as ‘Reovirus’. Detail information was collected in Supplementary Tables 1, 2, from which the figure was generated using Python (Matplotlib).

## The start: clinical case reports of tumor regressions after virus infection

The therapeutic potential of viruses in oncology was initially inferred from clinical observations of spontaneous tumor regression following naturally acquired viral infections. Notably, transient remissions in patients with leukemia and lymphoma were historically reported during concurrent influenza or measles virus (MV) infections. More recently, similar remissions have been documented in hematologic malignancies following severe acute respiratory syndrome coronavirus 2 (SARS-CoV-2) infection. The inherent tumor-selectivity demonstrated by many wild-type viruses is largely attributed to the altered cellular landscape of neoplastic cells. Specifically, the hallmarks of cancer create a permissive microenvironment that overlaps significantly with cellular features required for efficient viral replication.

## Oncolytic viruses: a new generation of live cancer cell killers

OVs are replication-competent, biologically active agents that exert anti-cancer effects through two principal mechanisms: direct tumor cell lysis and indirect stimulation of systemic anti-tumor immunity. In hematologic malignancies, where cancer cells reside in immune-privileged niches such as the bone marrow or peripheral blood, this dual functionality is particularly advantageous. On one hand, OVs themselves exploit molecular vulnerabilities in malignant cells; on the other hand, OVs can be armed with immunomodulatory transgenes to amplify their therapeutic impact.

## Pre-clinical studies on evaluating therapeutic OVs in blood cancers

To comprehensively examine and summarize the existing literature regarding OVs in blood cancer therapy and explore treatment-related outcomes, we first retrieved citations using the terms indicated in the Methodology section. After the title and abstract screening phase, the non-overlapping research articles were combined and analyzed. Then we grouped these studies according to the different virus types. Among them, MV was the most frequently studied in publications, followed by adenovirus, vesicular stomatitis virus (VSV) and reovirus (Figure 1 and Supplementary Table 1). Below, we will analyze the study setup and major outcomes of these studies based on different OV types.

Although adenovirus was the most frequently applied one in clinical trials,^1^ only a handful of studies were performed in blood cancer treatment. These studies ranged from systematic evaluation to rational design of human adenoviruses for enhanced blood cancer therapy. In an earlier study by Qian and colleagues, E1B55K-deleted adenovirus type 5 (Ad5) armed with melanoma differentiation-associated gene-7/interleukin-24 (MDA-7/IL-24) was tested in a range of blood cancer cell lines. Although a potent anti-cancer effect was observed *in vitro* and *in vivo,* the effect was limited to Ad5 major receptor (coxsackievirus and adenovirus receptor)-expressing hematological malignancies (Qian et al., 2008). Follow-up studies solved the problem by using chimeric Ad35 fiber (Wang et al., 2009; Wenthe et al., 2020). Another study compared the growth-inhibition effect of adenovirus and herpes simplex virus (HSV) on several cell lines; HSV was more effective (Zhang et al., 2011). Realizing the limitation from Ad5, a broad range of human adenoviruses were evaluated by Barry’s research group. They observed robust killing and delayed lymphoma growth by species D adenoviruses (Chen et al., 2011). However, in mouse B cell lymphoma model A20, only prophylactic immunization by adenovirus against A20 tumors can protect against A20 lymphomas; established A20 tumors were not controlled by A20-expressing adenovirus immunization (Weaver et al., 2011). While OV alone is not enough to control blood malignancies, a combination approach highlights the optimized development strategy. A rationally designed OAd5F35, Ad5-based chimeric virus with Ad35 fiber, armed with TMZ-CD40L and 4-1BBL was evaluated in combination with CAR-T therapy. The fiber switch to Ad35 enabled the use of CD46, which is up-regulated in hematological malignancies, thereby enhancing targeting and replication. Interestingly, OAd5F35 infection induced transgene expression and a change in immune markers. In combination with CAR-T therapy, the infected lymphoma cells were found to secrete chemokines to attract CAR-T cells, thereby increased CAR-T cell activation was observed. Importantly, the enhanced lymphoma cell killing was also observed *in vivo* in xenograft models (Wenthe et al., 2021). Similarly, a CD47 antibody expressing adenovirus (Ad5-hTert-E1A-IRES-E1B with mCMV-mCD47nb-Fc) as *in situ* tumor vaccination also showed positive immune-regulation and protection from tumor re-challenge (Zhang et al., 2022). These data suggested that adenovirus alone is not enough to control such aggressive hematological malignancies; a combination approach should be the development strategy of choice.

Talimogene laherparepvec (T-VEC), based on HSV-1, is so far the only OV approved by the U.S. Food and Drug Administration (FDA) and the European Medicines Agency (EMA) for the treatment of melanoma (Zhang T. et al., 2023). However, only a few studies have explored its potential in hematological malignancies. In the search for molecular indicators for permissiveness to HSV therapy, transcription factors downstream of Ras were evaluated in non-Hodgkin’s lymphoma (NHL) cell lines infected with R3616, an HSV-1 with a deletion of the viral γ134.5 gene. Ras signaling was assessed by immunoblotting for different downstream effectors, which revealed ERK/ELK as a suitable predictive indicator for selection of cancer cells with increased sensitivity to HSV-based therapy (Esfandyari et al., 2009). As G47Δ was approved in Japan in 2021 (Frampton, 2022), a similar version of this OV was evaluated in a broad panel of 26 cell lines and 15 primary cells derived from various lineages of hematological malignancies (Ishino et al., 2021). The expression of an HSV-1 receptor, nectin-1, was identified as a determining factor in the oncolytic effect determined by viral entry and cytotoxicity. Although only a low level of virus replication was detected in hematological cancer cells, the virus could induce immunogenic cell death and kill the malignant cells. Importantly, this study demonstrated that systemic tumor-specific CD8 + T cell responses efficiently concentrated at tumor sites, suggesting that local intratumoral injection of HSV-1 is expected to provoke effective immune responses even for systemic tumors such as hematological malignancies. Recently, an oncolytic type II HSV (oHSV2) was explored in the treatment of diffuse large B-cell lymphoma (DLBCL) (Zhang J. et al., 2023). In comparison with the clinical chemotherapeutic drug doxorubicin (DOX), oHSV2 revealed a better therapeutic profile on the apoptotic, invasive, and proliferative capacity of DLBCL cells *in vitro*. Moreover, oHSV2 can also inhibit transplanted tumor growth and accelerate CD4 + T and CD8 + T cell infiltration in mice, with an acceptable safety profile in the organs of tumor-bearing animals. To explore the potential of combination therapy with immune-checkpoint inhibitors, this study also showed oHSV2 can inhibit the PD-1/PD-L1 immune-checkpoint, thereby restraining cytotoxic T lymphocyte (CTL) apoptosis and encouraging CTL proliferation. The combination therapy resulted in enhanced killing of malignant cells and inhibited tumor growth in mice. Similar to the adenovirus studies, combination therapy and the individual receptor profile are essential for further HSV application in hematological malignancies.

The rat protoparvovirus H-1 (H-1PV) does not infect or cause any disease in humans, but has natural tropism for human cancer cells. H-1PV demonstrates a broad range of anticancer activities in several preclinical cancer models and in clinical trials (Geletneky et al., 2017; Bretscher and Marchini, 2019; Hajda et al., 2021). The first study to apply H-1PV to Burkitt’s lymphoma (BL)-derived cell lines showed high sensitivity to H-1PV-induced oncolysis *in vitro*. The H-1PV infection of BL lines is productive and leads to predominantly necrotic cell death. Importantly, normal B cells from healthy donors are resistant to H-1PV infection (Angelova et al., 2009). Then a large panel of lymphoma- and leukemia-derived cell lines was included to assess the capacity of H-1PV to target hematological malignancies. This study revealed that most cell lines are sensitive to H-1PV-induced cell death, and the progeny virion production, rather than virus entry, was the determining factor. Among all tested cells, DLBCL cells supported high levels of H-1PV progeny virion production and were almost totally eradicated by very low virus doses. Moreover, DLBCL cell lines with upregulated expression of aldehyde dehydrogenase 1A1 conferring chemotherapy resistance were among the most sensitive H-1PV targets (Angelova et al., 2017). A recent study extended this work to cutaneous T-cell lymphoma (CTCL). H-1PV infects and induces oncolysis in both classically grown CTCL suspension cultures and in a novel, *in vivo*-relevant, heterotypic spheroid model, but not in healthy donor controls, including peripheral blood mononuclear cells (PBMCs). In CTCL spheroid co-cultures with PBMCs, treatment with the virus increased infiltration of the spheroids with immune cells (Angelova et al., 2024).

Among oncolytic platforms, MV is the most extensively studied in hematological malignancies, owing to its vaccine safety record and its natural tropism for CD46 and the lymphocyte receptor SLAM/CD150 (Ong et al., 2006; Engeland and Ungerechts, 2021). Vaccine-strain MV was found to regress human B-cell lymphoma xenografts even in the presence of anti-MV antibodies (Grote et al., 2001). Subsequent improvement in specificity was achieved by retargeting viral entry. Vectors retargeted to CD20, armed with a prodrug convertase, and combined with cyclophosphamide and fludarabine regressed mantle cell lymphoma xenografts, though with limited survival benefit (Bucheit et al., 2003; Ungerechts et al., 2010). Analogous CD30 and CD38 retargeting extended the approach to Hodgkin lymphoma and myeloma (Peng et al., 2003; Hanauer et al., 2018). Importantly, efficacy was tracked with receptor usage and innate signaling. For instance, SLAM-dependent entry in mantle cell lymphoma virotherapy, EBV-driven CD150 upregulation and type I interferon responsiveness have been shown to determine susceptibility, and Bcl-2 has been shown to slow oncolysis in chronic lymphocytic leukemia (Patel et al., 2011; Miest et al., 2013; Fernandez et al., 2014; Takeda et al., 2014). To facilitate systemic delivery in the face of anti-MV immunity, studies have shown that both carrier and mesenchymal stromal cells have the capacity to deliver the virus to sites of disseminated disease (Iankov et al., 2007; Castleton et al., 2014). In particular, the development of the thyroidal sodium iodide symporter-expressing MV-NIS has advanced MV into early-phase clinical testing for multiple myeloma (MM) (Dingli et al., 2004).

Vaccinia virus (VV), a large-genome poxvirus that tolerates sizeable transgene insertions, has been used in blood cancers mainly as an arming and combination platform rather than as a bare oncolytic agent. An attenuated VV delivered by cytokine-induced killer cells trafficked to residual murine B-cell lymphoma and prevented relapse through acquired antitumor immunity (Contag et al., 2010). Its coding capacity has since carried both pro-apoptotic and immunomodulatory transgenes. Vectors co-expressing miR-34a and Smac have been shown to induce cell death in MM cells (Lei et al., 2016), and a VV-Beclin1 drove autophagic death of NHL cells, sensitizing them to R-CHOP immune-chemotherapy (Rituximab, Cyclophosphamide, Hydroxydaunomycin, Oncovin and Prednisone) (Xie et al., 2021). Notably, immune-arming constructs have been developed to combine oncolysis with a defined effector mechanism. A CD19 bispecific T-cell engager recruited T cells, an anti-CD47 nanobody reprogrammed macrophages, and interleukin-18 relieved T-cell exhaustion, each prolonging survival in lymphoma models (Lei et al., 2022; Li et al., 2025; Wu et al., 2026). The natural adjuvant embelin further enhanced replication and antitumor immunity (Wang et al., 2020). With its large coding capacity and systemic deliverability, vaccinia is a flexible platform whose efficacy comes mainly from immune arming, not oncolysis alone.

VSV, a non-human rhabdovirus highly sensitive to type I interferon, selectively replicates in malignant cells with defective antiviral signaling, and its development has balanced potency against inherent neurotoxicity. A particular line of research has identified a correlation between VSV and the apoptotic vulnerabilities of lymphoid malignancies. A cytosine deaminase/uracil phosphoribosyl transferase suicide gene produced a 5-fluorouracil bystander effect against A20 lymphoma (Porosnicu et al., 2003; Leveille et al., 2011b), BCL-2 inhibitors sensitized resistant chronic lymphocytic leukemia cells (Tumilasci et al., 2008; Samuel et al., 2013), and VSV-driven Mcl-1 degradation overcame chemoresistance (Schache et al., 2009). A secondary line engineered the backbone for safety and combination. VSV-IFNβ-NIS gained tumor specificity, reduced neurotoxicity, and imaging capability, achieving complete remission in syngeneic myeloma after a single intravenous dose (Naik et al., 2012), and acted synergistically with anti-PD-L1 in acute myeloid leukemia (Shen et al., 2016). Retargeting to CD4 selectively destroyed adult T-cell leukemia/lymphoma without neurotoxicity (Betancourt et al., 2015), although VSV can also infect tumor-associated dendritic cells and impair antigen presentation (Leveille et al., 2011a). In a comparative-oncology study, intravenous VSV-IFNβ-NIS induced remissions in dogs with spontaneous T-cell lymphoma and informed the clinical translation of the construct (Naik et al., 2018). The insertion of IFN-β and the retargeting to CD4 were both intended to limit VSV’s inherent neurotropism. In the Phase I trial of VSV-hIFNβ-NIS, a single intravenous dose caused no dose-limiting toxicities and no reported neurotoxicity; the main adverse event was transient grade 1–2 cytokine release syndrome (Cook et al., 2022).

Reovirus is a naturally oncolytic double-stranded RNA virus that needs no genetic engineering and has reached clinical trials as pelareorep. In blood cancers, susceptibility depends on the junctional adhesion molecule-A (JAM-A) rather than Ras; JAM-A is overexpressed on multiple myeloma cells and rises further at relapse and under bortezomib resistance (Kelly et al., 2015). Reovirus killed myeloma cells while sparing hematopoietic stem cells, and has therefore been used ex vivo to purge autologous stem-cell grafts (Thirukkumaran et al., 2012; Thirukkumaran et al., 2014). Because the virus often enters myeloma cells but replicates poorly, most studies combined it with sensitizing agents such as proteasome inhibitors, histone deacetylase inhibitors or NAD + depletion to increase endoplasmic reticulum stress and apoptosis (Kelly et al., 2012; Stiff et al., 2016; Kennedy et al., 2022). Beyond direct lysis, reovirus activated natural killer and cytotoxic T cells that killed myeloma within the protective bone-marrow niche and improved rituximab-mediated killing of chronic lymphocytic leukemia, whereas resistance in HTLV-1-transformed T-cell lymphoma was overcome by JAK/STAT inhibition (Parrish et al., 2015; Islam et al., 2021; Muller et al., 2021). It was similarly effective against mantle cell lymphoma and acute myeloid leukemia, where mesenchymal stromal cells and their exosomes delivered the virus past neutralizing antibodies (Alain et al., 2010; Hall et al., 2012; Yang et al., 2024).

Beyond these platforms, a diverse group of viruses has been evaluated in the context of blood cancers. Several are non-human agents chosen to avoid pre-existing human immunity. Newcastle disease virus (NDV) demonstrated early preferential toxicity towards BL in comparison to normal lymphocytes (Tzadok-David et al., 1995; Bar-Eli et al., 1996). Attenuated strains later led to the selective elimination of human, canine, and feline lymphoma cells, inducing apoptosis and acting synergistically with rituximab and doxorubicin (Sanchez et al., 2015; Al-Shammari et al., 2016; Mohammed et al., 2019; Alves et al., 2025). Myxoma virus was initially tested for purging MM cells from ASCT grafts and was subsequently shown to induce ligand-independent caspase-8 apoptosis in myeloma cells, thereby preventing the development of post-transplant EBV lymphoma (Bartee et al., 2012; Kim et al., 2015; Bartee et al., 2016). Similarly, bovine viral diarrhea virus has been shown to eliminate CD46 + myeloma cells, with the addition of bortezomib resulting in a synergistic effect (Marchica et al., 2020). In contrast, others are human viruses that possess intrinsic oncolytic tropism. Coxsackievirus A21 has demonstrated efficacy in lysing CD138 + plasma cells in patient bone marrow biopsies and drug-resistant B-cell malignancies, while sparing progenitor cells and healthy B cells (Au et al., 2007; Holmes et al., 2023). Sindbis vectors were evaluated mainly in murine lymphoma, curing A20 when combined with an anti-4-1BB antibody (Suzme et al., 2012; Yu et al., 2019). Most of these agents remain at an early, single-study stage, but the strategies they illustrate, using non-human viruses to sidestep antiviral immunity and delivering virus ex vivo or by carrier cells, are directly relevant to the more advanced platforms above.

Although OVs show promising therapeutic effects in blood cancers, there are not yet enough data that directly compare the efficacy of different OVs or validate the therapeutic outcomes of a specific virotherapeutic treatment, as in other cancer types (Schafer et al., 2024). The only study that compared adenovirus and HSV was based solely on Ad5, which has a certain receptor-limitation (Zhang et al., 2011). Therefore, our current literature analysis highlights the need for a broad laboratory evaluation involving more OV types.

Regarding the delivery strategies in hematologic malignancies, two major strategies for OV delivery were explored—local (intratumor) and systemic (intravenous or subcutaneous) administration. Intratumor OVs administration offers advantages of higher virus dose at the lesions with better effectiveness, while minimizing systemic exposure and side effects elsewhere in the body. Which is prefer used in solid tumor. For metastatic and widespread cancers and blood cancers, systemic administration is advantageous. However, systemic delivery faces challenges including neutralizing antibodies, complement activation, and hepatic clearance. To address this, novel strategies include capsid engineering (e.g., Ad5F35 or Ad5F3 chimeras), use of non-human viruses such as vesicular stomatitis virus (VSV) or myxoma virus and carrier-cell systems where autologous immune or stromal cells transport the virus to tumor sites (Wang et al., 2009; Havunen et al., 2017; Villa et al., 2022). In leukemias, emerging techniques such as intramedullary injection or OV-modified stem cell grafts aim to increase delivery efficiency directly within the bone marrow were also explored (Castleton et al., 2014).

## Clinical trials and case reports using OVs to treat blood cancers

As presented in the previous sections, extensive efforts have been devoted to the development of OVs for the treatment of hematopoietic malignancies. Although preclinical studies have demonstrated promising results, clinical trials remain essential to establish their safety and therapeutic efficacy in patients. In contrast to the large body of preclinical research, only a limited number of OVs have been evaluated in clinical trials for hematologic cancers, as summarized in Figure 1 and Supplementary Table 2. The clinical landscape is currently dominated by early-phase (Phase I) dose-escalation studies aimed at establishing the maximum tolerated dose (MTD) and safety profiles, supplemented by strong translational efficacy signals from case reports and observational data demonstrating synergistic oncolysis and immune activation.

The core of ongoing clinical research focuses on evaluating the safety and preliminary efficacy of viral vectors, frequently utilizing standard 3 + 3 dose-escalation methodologies. The VSV-hIFNβ-NIS construct has yielded the most actionable Phase I clinical data. Evaluated for relapsed/refractory lymphomas, acute myeloid leukemia (AML), and MM, the virus is administered both as a monotherapy and in combination with cyclophosphamide and checkpoint inhibitors (ipilimumab, nivolumab, cemiplimab) (NCT03017820). Notably, 3 out of 7 patients with T-cell lymphoma exhibited objective responses, including partial remissions at 3 and 6 months, and an ongoing durable complete remission exceeding 20 months in a heavily pretreated cohort. Meanwhile, multiple VV constructs are under investigation to target specific lymphoid lineages. RT-01 utilizes an intravenous administration schedule (Days 1 and 6, followed by 8-week maintenance cycles) for T-cell lymphoma (NCT05387226). For B-cell malignancies, RGV004 and RGV005 are evaluating MTD and dose-limiting toxicities, with RGV005 comparing intratumoral versus intravenous injection routes across four distinct dose cohorts. In addition, reovirus (Pelareorep) is being evaluated for relapsed MM in multi-arm studies to determine optimal dosing when administered intravenously alongside standard anti-neoplastic regimens (carfilzomib, nivolumab, and dexamethasone) (NCT03605719). Two separate clinical trials showed that the combination of Pelareorep with a proteasome inhibitor can have an immune-activating effect towards cancerous cells of MM. In the first trial (NCT02101944), 13 heavily pretreated patients received carfilzomib, dexamethasone, and Pelareorep, with biopsies confirming active viral replication inside myeloma cells. This treatment also induced monocytic expansion, CD8 + T cell activation, caspase-3 activation indicating cell death, and increased PD-L1 expression. In the second trial (NCT02514382), 14 patients received bortezomib, dexamethasone, and Pelareorep, leading to decreased paraprotein in six patients. Additionally, this second regimen increased T and NK cell activation, inflammatory cytokine release, and bone marrow PD-L1 expression.

Meanwhile, case studies and observational frameworks provide critical proof-of-concept for viral-mediated cytotoxicity and chemo-sensitization. For example, intravenous infusion of high-dose MV-NIS in two measles-seronegative patients with drug-refractory MM resulted in robust clinical responses (Russell et al., 2014). Both patients demonstrated significant monoclonal protein reduction and clearance of bone marrow plasmacytosis, with one achieving complete and durable remission across all disease sites. In a complex case of adult T-cell leukemia/lymphoma, a localized intervention utilizing T-VEC injections combined with mogamulizumab and 4 Gy low-dose radiation induced direct oncolysis (An et al., 2025). This multimodal approach effectively reduced lesion size, stabilized the disease state, and extended patient survival by 5 months prior to terminal progression. In another study, T-VEC administered via intratumoral injection for primary cutaneous B-cell lymphoma induced positive tumor responses in 11 out of 13 patients, with 6 patients achieving a complete response (Ramelyte et al., 2021). This regression correlated with a localized and systemic reduction in B-cells alongside an infiltration of NK-cells and T-cells, indicating a targeted, virus-mediated immune activation.

A Phase I trial utilizing VSV-hIFNβ-NIS against relapsed/refractory MM, T-cell lymphoma (TCL), and AML demonstrated a dose-dependent objective response, with a complete response in one TCL patient (Cook et al., 2022). Observed toxicities were limited to transient events such as low-grade cytokine release syndrome. Further case studies reported that natural SARS-CoV-2 infection was followed by transient remission or tumor reduction during periods when standard chemotherapy was delayed. In cases of AML and T-cell acute lymphoblastic leukemia (ALL), blast cell counts decreased significantly during acute infection (Kandeel et al., 2021). Comparable clinical outcomes—including the resolution of pathological lymphadenopathy and reductions in malignant NK-cell populations—were documented in high-grade B-cell lymphoma and refractory NK/T-cell lymphoma (Pasin et al., 2020; Yilmaz et al., 2021). These clinical observations indicate that SARS-CoV-2 exhibits intrinsic oncolytic properties, functioning either through direct tumor cell lysis with subsequent antigen release, or through the induction of immune cross-reactivity against the underlying malignancy.

In summary, the aggregation of current data suggests that while monotherapy OVs demonstrate isolated efficacy, the highest therapeutic index is achieved via multi-mechanistic combination therapies. Integrating OVs with systemic immunotherapies (checkpoint inhibitors, monoclonal antibodies) or established chemotherapeutic and radiotherapy regimens presents a highly viable, synergistic strategy to bypass multidrug resistance in refractory hematopoietic malignancies. Furthermore, natural concomitant infections of cancer patients, e.g., with SARS-CoV-2, should be studied thoroughly. Spontaneous remission in cancer patients seems to be mediated by certain predictable pathways. Studying and understanding the mechanism behind these favorable outcomes might help to create new, more efficient OVs.

## Future perspectives: take DLBCL as targeting example

While OVs show promise across hematologic malignancies, these diseases reside in distinct anatomical niches that impose unique physical and immunological barriers to viral therapy. For instance, the bone marrow niche (predominant in MM and AML) exhibits elevated interstitial pressure and dense stromal packing that impede viral penetration (Gastelum et al., 2021; Pimenta et al., 2021). Furthermore, hypoxia-inducible factors (HIFs) within the bone marrow can alter virus entry receptors (e.g., CD46, CAR, nectin-4, integrins) on malignant cells (Basheeruddin and Qausain, 2024). In contrast, lymph node microenvironments in diseases like DLBCL present distinct barriers: while lymphatic drainage facilitates access, dense lymphocytic architecture and abundant plasmacytoid dendritic cells (pDCs) trigger strong local type-I interferon signaling upon viral exposure, rapidly establishing a potent antiviral state that can restrict viral spread (Li et al., 2022). Understanding these niche-specific differences in viral access, receptor regulation, and innate immune sensing is essential for designing tailored virotherapeutic strategies.

Among hematopoietic malignancies, DLBCL is the most common and aggressive type, accounting for 30–40% of new lymphoma cases, with ~150,000 newly diagnosed cases annually. Although two-thirds of DLBCL patients can be cured with the frontline R-CHOP immuno-chemotherapy, for the remaining one-third of patients with refractory or relapsed disease following the first-line therapy, treatment remains challenging with poor outcomes due to the aggressive nature of the disease (Sehn and Salles, 2021). Therefore, virotherapy, which has been shown to induce remission of leukemias and malignant lymphomas following natural virus infections, should be re-discovered and play its role in lymphoma. DLBCL serves as an ideal clinical model to illustrate the future of next-generation OV design. As an aggressive and highly heterogeneous malignancy, DLBCL highlights the precise engineering hurdles that OVs must overcome to transition from bench to bedside. The future roadmap for optimizing OVs against DLBCL points to three strategies: (1) enhance the targeting mechanics, (2) arm the OVs with immunostimulatory factors, and (3) rationally combine them with other regimens.

As shown in studies with CD20, CD30, or CD38-targeted MVs, envelope pseudotyped with single-chain variable fragments (scFv) or designed ankyrin repeat proteins (DARPins) targeting canonical B-cell markers such as CD19, CD20, CD22 or BCMA (CD269) is a rational strategy to bypass native viral receptors and eliminate off-target sequestration in healthy tissues (Bucheit et al., 2003; Peng et al., 2003; Hanauer et al., 2018). Meanwhile, as DLBCL frequently exhibits antigen escape and phenotypic shifting under standard therapeutic pressures (Wang et al., 2022; Zhang et al., 2026), dual-targeted OVs, like the CD20-CD19 bispecific CAR-T cells recognizing both CD19 and CD20, should be considered (Martyniszyn et al., 2017).

Secondly, future research should also focus on engineering OVs with enhanced immunostimulatory capacity. Genetic modification of OVs to express cytokines such as GM-CSF, IL-12, or IFN-β could amplify antitumor immune responses and overcome the immunosuppressive milieu characteristic of aggressive B-cell lymphomas (Harrington et al., 2019; Hemminki et al., 2020; Gujar et al., 2024). In DLBCL, where defects in antigen presentation and T-cell exhaustion are common, using OVs as cancer vaccines to induce immunogenic cell death may increase tumor visibility to the immune system and improve responsiveness to immune checkpoint blockade (ICB) (Ahmed and Tait, 2020; Chang et al., 2023; Sousa-Pimenta et al., 2023; Jing et al., 2025).

Importantly, combination approaches represent the most promising clinical direction. OVs could synergize with rituximab, bispecific antibodies (BiTEs), CAR-T therapy, or immune checkpoint inhibitors (ICBs) by increasing immune-cell recruitment and promoting inflammatory remodeling of the tumor microenvironment. In particular, sequential administration of OVs before ICB or CAR-T therapy may enhance antigen release and T-cell infiltration, potentially improving CAR-T efficacy in patients with resistant disease (Ajina and Maher, 2017; Wenthe et al., 2021; Matsuzaka and Yashiro, 2025). Moreover, advances in precision medicine may allow the selection of viral platforms according to the molecular subtype of DLBCL, including germinal center B-cell–like (GCB) and activated B-cell–like (ABC) subtypes, each characterized by distinct signaling pathways and antiviral responses (Chapuy et al., 2018; Sehn and Salles, 2021).

While combining oncolytic viruses with CAR-T therapy, BiTEs, and ICBs offers synergistic potential, it simultaneously elevates the risk of severe systemic toxicities, including Cytokine Release Syndrome (CRS), Immune Effector Cell-Associated Neurotoxicity Syndrome (ICANS), and prolonged bone marrow aplasia (Rezaei et al., 2022; Feng et al., 2025). Oncolytic virus-induced immunogenic cell death and localized pathogen-associated molecular patterns (PAMPs) can amplify pro-inflammatory cytokine cascades, potentially worsening CAR-T or BiTE-associated hyper-inflammation. Consequently, clinical translation requires cautious optimization—such as staggered dosing schedules, the incorporation of tightly regulated viral safety switches, and the proactive deployment of mitigation strategies like anti-IL-6 receptor antibodies to safely balance enhanced anti-tumor immunity with patient safety (Watanabe et al., 2021; Morita et al., 2024).

In summary, taking DLBCL as a targeting example demonstrates that the comprehensive toolkit for oncolytic virotherapy must evolve beyond simple tumor lysis. The future of lymphoma-targeted OVs relies on building smart, multi-mechanistic platforms that seamlessly integrate precision surface targeting, localized microenvironment remodeling, and strategic synergy with cellular therapies.

## Footnotes

1
https://clinicaltrials.gov/
