# Supplementary material for: Therapeutic potential of oncolytic viruses in hematopoietic malignancies: from preclinical insights to clinical realities
Source: Front Microbiol. 2026 Sep 29;17:1925346. doi: 10.3389/fmicb.2026.1925346 (PMC13659887; doi:10.3389/fmicb.2026.1925346)
Supplement: Supplementary file 1 [file Table_1.docx]

**Supplementary table 1.** Pre-clinical studies of oncolytic viruses for blood cancer therapy.

| **Virus constructs** | **Cancer type /Tumor target** | **Therapy Strategy /aim of the study** | **Achievement /outcome** | **Reference** |
| --- | --- | --- | --- | --- |
| **Adenoviruses, conditionally replicating adenoviruses (CRAds)** | | | | |
| ZD5: Ad5-dE1B55K;  ZD55-IL24;  ZD55-EGFP  fgAd-IL24 (dE1) | -Mononuclear cells (MNCs) from patients bone marrow samples  -human leukemia cell lines (Molt-4, L428, K562) human myeloma cell line RPMI 8226  Kasumi and Mutz-1 cell line | E1B55K-deleted Ad5 expressing melanoma differentiation-associated gene-7/interleukin-24 (MDA-7/IL-24);  Concomitant with upregulation of RNA-dependent protein kinase R (PKR), increased phosphorylation of p38 mitogen-activated protein kinase (MAPK), and induction of endoplasmic reticulum (ER) stress. | -Only in CAR-expressing hematological malignancies  -exerted a potent antileukemia effect in vitro and in vivo | (Qian et al., 2008) |
| SG235: Ad5F35-dE1B55K | Human B-lymphoblastic leukemia cell line Mutz-1;  Human lymphoma cell line L428;  Human myeloma cell line RPMI 8226 | To overcome the Ad5 major receptor limitation | significantly suppressed malignant B-cell growth in vitro and in vivo;  induced apoptosis in hematopoietic malignant cells;  Enhanced antitumor activity in subcutaneous Mutz-1 xenografts | (Wang et al., 2009) |
| SG235-TRAIL + homoharringtonine (HHT) | Leukemia (Kasumi-1, KG-1, HL-60, U937) | Combine oncolytic Ad-TRAIL with HHT | HHT synergized cytotoxicity (↑apoptosis, caspase-3/9; ↓Bcl-2/Mcl-1/Bid); no significant toxicity to normal MNC/MSC | (Meng et al., 2009) |
| AdEHCD40L (conditionally replicating Ad expressing CD40L) | Multiple myeloma (RPMI 8226, Kas-6/1; chemoresistant MR20/LR5); SCID/hu | MM-targeted CD40L by oncolytic Ad under hypoxia | Inhibited MM growth more than control (CD40L-dependent); apoptosis + S-phase block, ↑TRAIL/Fas/IL-8; i.t. reduced xenograft >50% | (Fernandes et al., 2009) |
| SG611-PDCD5: triple-regulated oncolytic adenovirus carrying the pro-apoptotic PDCD5 gene | Leukemia cell lines; xenograft | Arm a triple-regulated (tumor-selective) oncolytic adenovirus with PDCD5 to enhance apoptosis of leukemic cells | -Selective replication in leukemic cells  -PDCD5 arming induced potent apoptosis and antitumor efficacy in vitro and in vivo | (Xie et al., 2009) |
| SG235-TRAIL (Ad5F35 CRAd expressing TRAIL) | Leukemia (CAR-low); normal progenitors spared | CAR-independent entry + TRAIL arming | Selective cytopathic effects; apoptosis via extrinsic+intrinsic pathways; superior antileukemia vs SG235; progenitors resistant | (Jin et al., 2009) |
| SG235 (Ad5F35 chimeric CRAd) | Leukemia lines; CD46+ patient blasts | Evaluate SG235 infection/cytotoxicity (CD46-dependent) | Efficiently infected CD46+ leukemia (GFP 17–67%); growth inhibition and apoptosis of Kasumi-1; inhibited p-Akt | (Yang et al., 2010) |
| Ad5 and low-seroprevalence Ads (Ad6, Ad11, Ad26, Ad35, Ad48) | Multiple myeloma (lines, primary CD138±) | Compare adenovirus serotypes for MM oncolysis | Ad5/Ad6 (C) and Ad26/Ad48 (D) killed CD138+ MM with replication; Ad11/Ad35 bound but didn't replicate/kill | (Senac et al., 2010) |
| HSV-1-GFP;  HSV-1-GFP-delIRs (BH-1-G-dirs);  Adv-GFP | Human BL cell line Raji, TL cell line Jurkat, cutaneous TL cell line Hut 78, NK/T lymphoma cell line SNK-6 | evaluate 3 recombinant viruses,  the efficiency of viral infection and replication and cytotoxicity to  4 types of lymphoma cells | HSVs infect and replicate in the three lymphoma cell lines (Jurkat, Raji and Hut 78) and inhibit the growth of these cells more efficiently than Adv-eGFP;  the human NK/T lymphoma cell line SNK-6 was not infected by any of the viral vectors and did not allow replication of the viruses | (Zhang et al., 2011) |
| Ad4, Ad5, Ad6, Ad7, Ad11, Ad17, Ad24, Ad26, Ad28, Ad30, Ad35, Ad45, and Ad48 | Mouse myeloma: 5TGM1  human Burkitt’s lymphoma: Raji  Human lymphoma: RL  Human B-CLL: Mec-1 and Mec-2  Patient samples: CLL, lymphoma, multiple myeloma, | Evaluate 15 Ads represent Ad species B, C, D, E, and F in cell lines and primary patient B-cell cancers for infection, replication in, and killing. | Species D Ads mediated most robust killing;  select species D viruses Ad26 and Ad45 delayed lymphoma growth. | (Chen et al., 2011) |
| Ad4, Ad5, Ad6, Ad17, Ad24, Ad26, Ad28, Ad30, Ad35, Ad45, and Ad48  Ad-A20: fgAd5 express A20-scFv  Ad-GL: fgAd5 express GFP/luciferase | Mouse B cell lymphoma: A20 | first-generation Ad5 expressing the A20 immunoglobulin ScFv immunogen (Ad-A20) as cancer vaccine | Prophylactic immunization by Ad against A20 tumors protected against A20 lymphomas;  Established A20 tumors are not controlled by Ad-A20 immunization;  species D Ad26 and Ad48 were effective to control A20 tumor growth immunocompetent mouse | (Weaver et al., 2011) |
| SG511-BECN (Ad5/11 oncolytic Ad expressing Beclin-1) | Leukemia (CML/AML lines, primary; imatinib/chemo-resistant) | Arm Ad with Beclin-1 for autophagic death of resistant leukemia | Autophagic cell death, enhanced killing (incl. resistant); spared normal cells; prolonged survival, reduced xenograft size | (Tong et al., 2013) |
| Ad.IL3-MnSOD  Ad.IL3-PPA (Ad redirected to CD123 via sCAR-IL3) | Acute myeloid leukemia (HL60, KG-1; HL60/Luc NOD/SCID) | Retarget Ad to CD123 + therapeutic arming | Infected AML via CD123; armed versions suppressed proliferation, induced caspase-9/-7 apoptosis, reduced burden, prolonged survival | (Li et al., 2014) |
| ZD55-TRAIL (oncolytic Ad expressing TRAIL) + LY294002/MG132 | Multiple myeloma (sensitive and resistant; RPMI-8226) | Combine Ad-TRAIL with PI3K or proteasome inhibition | More cytotoxic than virus alone via caspase apoptosis; LY294002 blocked virus-induced Akt/mTOR; MG132 ↑DR5 enhancing apoptosis without normal-liver toxicity | (Tong et al., 2014) |
| Ad.4N1-IL24 (Ad redirected to CD47 via sCAR-4N1, armed with mda-7/IL-24) | CD47+ leukemia (HL60/Luc xenograft) | Retarget Ad to CD47 + IL-24 arming | 4N1-CD47-mediated infection/cytotoxicity; Ad.4N1-IL24 replicated faster, enhanced antileukemia effect in vitro/in vivo | (Li et al., 2015) |
| AdCN205-11-IL-24 (chimeric Ad expressing IL-24) | Chronic myeloid leukemia | Chimeric Ad + IL-24 for CML | High hIL-24 in CML; specific cytotoxicity sparing normal cells; remarkable antitumor activity via apoptosis | (Wei et al., 2015) |
| AdCN205-11-IL-24 (Ad5/11 chimeric Ad expressing IL-24) | Acute myeloid leukemia | Ad5/11 fiber + IL-24 arming | High hIL-24; AML-specific cytotoxicity sparing normal cells; higher antitumor activity via apoptosis vs Ad5-based version | (Wei et al., 2015) |
| Triple-regulated oncolytic adenovirus carrying VSTM1 + daunorubicin | Leukemia cell lines; xenograft | Combine a triple-regulated oncolytic adenovirus armed with VSTM1 with daunorubicin | -Synergistic antitumor activity against leukemia cells  -Enhanced apoptosis and chemosensitization vs monotherapy | (Zhou et al., 2016) |
| SG611-PDCD5 (triple-regulated oncolytic adenovirus carrying PDCD5) + low-dose daunorubicin | Leukemic cell lines (K562, MEG-01, KG-1a, HL-60, SUP-B15, BV-173); K562 nude-mouse xenograft | Combine oncolytic Ad-PDCD5 with low-dose daunorubicin to lower chemo dose/toxicity | -Synergistic anti-proliferative and pro-apoptotic effects (Chou-Talalay synergy)  -Combination reduced xenograft tumor size and increased apoptosis vs either agent alone  -Chemosensitization to daunorubicin | (Zhou et al., 2018) |
| SG511-BECN (oncolytic Ad expressing Beclin-1) + doxorubicin | Multidrug-resistant CML (K562/A02) | Combine autophagy-arming Ad with doxorubicin | Improved antileukemia activity via autophagic death; doxorubicin synergized by improving infectivity, without significant normal-MNC damage | (Li et al., 2018) |
| AdCN306-IL-24 (Ad11-tropism chimeric Ad expressing IL-24) | Acute promyelocytic leukemia (APL) | CAR-independent (Ad11) entry + IL-24 for APL | Strong APL-specific cytotoxicity; IL-24 upregulated; cytolysis via programmed cell death | (Liu et al., 2019) |
| OAd5F35-CD40L-4-1BBL | Multiple myeloma | dysregulated retinoblastoma protein pathway is frequently observed in MM | All cells were sensitive to infection, leading to viral replication and cell killing  downregulate of markers connected to MM progression (ICAM-1, CD70, CXCL10, CCL2, and sIL-2Rα) and an upregulate of the death receptor Fas;  Promoted activation of cytotoxic T cells | (Wenthe et al., 2020) |
| OAd5F35-CD40L-4-1BBL | B-cell lymphoma cell lines (BC-3, Karpas422, Daudi, DG-75, U-698) | OAd5F35 armed with TMZ-CD40L and 4-1BBL combine with CAR T-cell therapy | OAd5/35 target and B‑cell lymphoma cell lines, and replicate even in cells more resistant to killing;  The infection induces transgene expression and a change in immune markers;  Infected lymphoma cells secrete chemokines and attract CAR T cells, thereby Increased CAR T‑cell activation;  Enhanced lymphoma cell killing also in vivo in xenograft models | (Wenthe et al., 2021) |
| oAd-mCD47nb-Fc:  Ad5-hTert-E1A-IRES-E1B with mCMV-mCD47nb-Fc | A20 (murine B-cell lymphoma) | -direct oncolysis  -elicit potent and durable antitumor immune responses by induction of immunogenic cell  death of tumors  -OV expressed anti-CD47 antibodies to induce potentiated tumor killing | - Enhances the Phagocytic Function of Macrophages and Induces Immunological Cell Death In Vitro  -In Situ Tumor Vaccination Represses Tumor Growth in Murine Models  - Enhances Immune Infiltration Within the TME  - Reprograms Tumor-Associated Macrophages  - Stimulates T Lymphocytes Within the TME and Spleen  - Protects Mice From Tumor Re-Challenge | (Zhang et al., 2022) |
| Ad[CE1A] (SLAMF7-promoter-driven oncolytic Ad) | Multiple myeloma (8 lines, 17 patients; 5TGM1, U266) | SLAMF7-restricted oncolytic Ad ± bortezomib/melphalan/panobinostat/pomalidomide | Selective MM oncolysis (sparing controls/HDs); caspase-independent (necroptosis); ↑ICD markers/MHC-I/II; reduced burden in vivo; melphalan ↑SLAMF7 boosting replication | (Stewart et al., 2025) |
| **Coxsackievirus A21 (CVA21)** | | | | |
| CVA21 | multiple myeloma (MM);  patient bone marrow (BM) biopsies | Assess the capacity of CVA21 to target and destroy MM and precursor aberrant plasma cells | tumor cells lines expressed high levels of CVA21 receptors (intercellular adhesion molecule-1 (ICAM-1) and decay-accelerating factor (DAF);  MM cell lines were remarkably susceptible to CVA21 lytic infection and replication;  Normal peripheral blood cells were refractile to CVA21 infection;  purging of up to 98.7% of CD138+ plasma cells in patient BM biopsies, without significant decrease in progenitor cell function | (Au et al., 2007) |
| CVA21 | Ramos, Raji, OCI-LY-19, SU-DHL-4, EHEB, and CD40L-L929 cell  lines and parental L929 cells | Test CVA21 in eradicating malignant B cells and drug-resistant disease | Healthy B cells are resistant to CVA21 oncolysis;  CVA21 induces direct oncolysis in a range of B cell malignancies;  B cell neoplasms show functional type I IFN response;  Drug-resistant B cells remain susceptible to CVA21-induced cell death: | (Holmes et al., 2023) |
| **Oncolytic type II herpes simplex virus (oHSV2); Oncolytic herpes simplex virus type 1 (HSV-1); G47delta, HSV-1 with ICP47 and ICP34.5 genes deletion, and deletion of the overlapping region of the a47 gene and the promoter of the US11 gene** | | | | |
| R3616: HSV-1 with deletion of viral g134.5 gene | NHL cell lines Daudi, Jurkat, NC37, Raji, Ramos and ST486 | evaluate the value of transcription factors down-stream of Ras as molecular indicators for permissiveness to HSV therapy | Raji and NC37 showed increase in the phosphorylation levels of ERK and ELK (a Ras effector pathway its down-stream transcription factor);  ATF2 (transcription factor down-stream of p38-kinase pathway) was activated in all studied cells.  Raji and NC37 cells were also most permissive cells to infection with  R3616, and the permissiveness was decreased upon treatment of cells with an inhibitor of ELK-DNA binding;  Suggest ERK/ELK as a suitable predictive indicator for selection of  cancer cells with increased sensitivity to R3616 | (Esfandyari et al., 2009) |
| Oncolytic HSV-1 and UV-inactivated HSV-1 (UV-HSV-1) | Leukemic cell lines and primary AML samples; healthy allogeneic lymphocytes as control; PBMC/NK | Use (UV-inactivated) HSV-1 to stimulate innate NK/PBMC antileukemic responses | -HSV-1 activated PBMCs to lyse leukemic lines and primary AML but not healthy lymphocytes  -UV-HSV-1 was equally effective and 1000-10,000x more potent at stimulating innate antileukemic responses  -NK-cell-mediated killing of leukemic cells | (Samudio et al., 2016) |
| T-01, with the same genome modification as G47delta | -Hematological Malignancies (human cell lines and patients derived primary cells)  -Murine T cell lymphoma cell line | Viral entry and the oncolytic effect were positively correlated with the expression level of nectin-1. | the expression of an HSV-1 receptor, nectin-1, is a determining factor in the oncolytic effect  -T-01 can Kill Human Cell Lines Derived from Various Lineages of Hematological Malignancies in vitro  - in vivo in an immunocompetent mouse model, intratumoral injection of T-01 into lymphoma induced regression of injected, as well as non-injected, contralateral tumors accompanied by abundant infiltration of antigen-specific CD8+ T cells. | (Ishino et al., 2021) |
| Oncolytic HSV-1 (oHSV-1) | Multiple myeloma (lines, primary); NECTIN-1/HVEM | Exploit HVEM/NECTIN-1 on MM | HVEM ubiquitously high on plasma cells; required for infection; oHSV-1 killed all MM lines via caspase-3 apoptosis (no replication); reduced tumor volume → HVEM a target | (Ghose et al., 2021) |
| oHSV-2 HG52 strain  With ICP47 and the ICP34.5 genes deletion | The human DLBCL cell lines SU-DHL-4 and SU-DHL-6 | -oHSV2 alone or combine with PD-1/PD-L1 inhibitors | -better therapeutic profile than the clinical chemotherapeutic drug doxorubicin (DOX) on the apoptotic, invasive, and proliferative capacity of DLBCL cells  - safety profile in the organs of tumor bearing mice  - mediate CD4 + T and CD8 + T cell infiltration in the lymphoma tumor microenvironment | (Zhang et al., 2023) |
| HSV1716 (SEPREHVIR; ICP34.5-deleted) ± bortezomib | Multiple myeloma (lines, primary; JJN-3 NSG; 5TGM1 syngeneic) | HSV1716 alone and with bortezomib; systemic models | Apoptosis (↑CASP1/8/9, BAX, BID, FASL); HSV1716+bortezomib prevented regrowth ~25 d; i.v. HSV1716 lowered tumor burden in both models | (Tazzyman et al., 2023) |
| Oncolytic bovine herpesvirus type 1 (BoHV-1; non-human) | Multiple myeloma (lines; patient BM cells); ± bortezomib/lenalidomide/daratumumab | Non-human herpesvirus to bypass anti-viral immunity + immune remodeling | Mitochondrial apoptosis, suppressed MYC/OXPHOS/UPR; ↑NK ligands, ↓MHC-I; selectively reduced malignant PCs/immunosuppressive myeloid; activated CD8/NK/monocytes; synergy with bortezomib/lenalidomide; ↑CD38 → daratumumab sensitization | (Raimondi et al., 2026) |
| **The rat protoparvovirus H-1 (H-1PV),** natural host are rats, is the smallest among all known OVs, with an overall diameter of the icosahedral capsid of about 18–26 nm and a genomic size  of about 5 kb | | | | |
| MVMp: minute virus of mice parvoviruses prototype strain | MAIDS: MuLV-induced lymphoma | To investigate the interaction of MVMp with lymphoid cells regarding both safety and possible immunomodulating  effects | besides tumor cells, rare lymphoid subpopulations—MDC and B1 lymphocytes, showed most parvovirus- driven gene expression in tumor-bearing animals | (Raykov et al., 2005) |
| Wt- H-1PV  H-1PV/EGFP | Several B cell lyphoma (BL) cell lines: Namalwa, Raji, and Elijah, RAMOS, Akata | apathogenic in humans  infect and suppress various rat  and human tumors in animal models | BL-derived cells lines are highly sensitive for H-1PV-induced oncolysis in vitro  Normal B cells from healthy donors are resistant to H-1PV infection  H-1PV infection of BL lines is productive and leads to predominantly necrotic cell death | (Angelova et al., 2009) |
| Wt- H-1PV  H-1PV/EGFP | A panel of lymphoma- and leukemia-derived cell lines | To assess the capacity of - H-1PV to target hematological malignancies derived cells | Most cell lines are sensitive to H-1PV-induced cell death;  progeny virion production, not virus entry, was the determine factor;  DLBCL cells supported high levels of H-1PV progeny virion production and were almost totally eradicated by very low virus doses;  DLBCL cell lines with upregulated expression of aldehyde dehydrogenase 1A1 conferring CHOP resistance were among the most sensitive H-1PV targets; | (Geletneky et al., 2017) |
| Wt- H-1PV  And H-1PV/EGFP | cutaneous T-cell lymphoma (CTCL) | H-1PV natural tropism for human cancer cells | -H-1PV infect and induce oncolysis in both classically grown CTCL suspension cultures and in a novel, in vivo-relevant, heterotypic spheroid model, but not in healthy donor controls, including peripheral blood mononuclear cells (PBMCs)  -CTCL spheroid co-cultures with PBMCs, increased spheroid infiltration with immune cells upon co-culture treatment with the virus | (Angelova et al., 2024) |
|  |  |  |  |  |
| **Measles virus (MV), Edmonston-B strain of measles virus (MV-Ed)** | | | | |
| MV-Ed and MVlacZ | B-cell lymphoma: DoHH2 and Raji cells | MV replication within the tumor /cells | -MV replicates lytically in DoHH2 and Raji cells in vitro;  -MV are capable of causing regression of large established lymphoma tumors in vivo;  -such effect is not abrogated by the presence of passively transferred anti-MV antibodies | (Grote et al., 2001) |
| MV-HαCD20: MV with H glycoprotein replaced by an H–anti-CD20 single-chain (scFv) fusion protein (± protease-cleavable linker)  control unmodified MV | CD20+ B-cell non-Hodgkin lymphoma  bilateral human tumour xenografts (CD20+ vs CD20−);  CHO and CHO-CD20 cells | Retarget MV entry to the CD20 antigen via an scFv–H fusion to gain B-NHL selectivity | -Modified H incorporated into virions as efficiently as wild-type H  -MV-HαCD20 replicated in CHO-CD20 but not parental CHO cells  -Systemic MV-HαCD20 retarded growth of CD20+ but not CD20− xenografts  -First proof that an scFv–antigen interaction can mediate oncolytic-virus entry and enhance in vivo activity | (Bucheit et al., 2003) |
| MV-aCD38 | multiple myeloma cell lines (ARH-77, JJN-3, RPMI 8226, MM-1, KAS 6/1);  PBLs | Re-targeted Mv to infect only myeloma cells to minimize collateral damage to normal tissues using scFv (plasma cell marker CD38) | Replication of MV-aCD38 was not hindered by the scFv;  CHO cells expressing CD38 became susceptible to infection by MV-aCD38;  MV-Edm and MV-aCD38 showed equal cytotoxic for myeloma cell lines and caused minimal cell death in non-transformed PHA-stimulated PBLs | (Peng et al., 2003) |
| MV-NIS | multiple myeloma cell line: KAS-6/1  Primary myeloma cells (CD138 enriched) | Image-guided radiovirotherapy | MV-NIS replicated almost as efficiently as unmodified MV-Edm;  regressed myeloma xenografts completely after a single intravenous dose,  MV-resistant MM1 tumors regressed completely combine with iodide | (Dingli et al., 2004) |
| Attenuated canine distemper virus expressing eGFP (CDV-eGFP); MV (Edmonston) as comparator | Canine lymphoid cell lines; neoplastic B and T lymphocytes from dogs with spontaneous lymphoma | Determine whether canine lymphoid cells can be infected by MV or CDV (canine Morbillivirus) and whether infection triggers apoptosis; establish the dog as a large-animal model for Morbillivirus therapy of NHL | -Canine lymphoid cells expressed CD150 (SLAM); only neoplastic cells expressed CD46  -MV did not infect canine cells, but CDV infected 40–70% of cell lines and 50–90% of primary neoplastic B/T lymphocytes  -CDV-infected cells underwent apoptosis  -Attenuated CDV proposed for canine lymphoma; dogs are a relevant model for human NHL Morbillivirus therapy | (Suter et al., 2005) |
| Live MV, Edmonston-Zagreb vaccine strain | Cutaneous T-cell lymphoma (CTCL); CTCL cell lines and patient tumours (CD150+/CD46+) | Phase 1 dose-escalation of intratumoral MV in CTCL (with IFN-α pretreatment to limit spread); evaluate cytopathic and immune effects | -16 intratumoral MV injections in 5 patients were well tolerated, with clinical responses  -Local viral activity (MV-NP+), increased IFN-γ/CD4 and IFN-γ/CD8 mRNA ratios and reduced CD4/CD8 ratio  -All patients showed rising anti-MV antibody titres  -CTCL (often IFN-signalling deficient) is a promising target for MV oncolytic therapy | (Heinzerling et al., 2005) |
| MV-eGFP | Primary myeloma samples | To evaluate CD46 levels on MM; | surface levels of CD46 on MM cells are correlated with their susceptibility to MV-induced cytopathic effects; | (Ong et al., 2006) |
| Recombinant MV vaccine strain expressing eGFP (rMV-eGFP) | CTCL; three patient-derived CTCL cell lines; established CTCL xenografts in nude mice | Characterise rMV cytolytic activity in CTCL cultures and xenografts; relate susceptibility to MV-receptor expression | -All CTCL lines expressed CD150 and CD46, were readily infected and completely lysed  -Cytoreduction via cell-to-cell fusion (aggregates)  -Intratumoral rMV-eGFP induced complete regression of large CTCL xenografts while controls progressed  -MV replication confirmed within tumours (MV-NP) | (Kunzi et al., 2006) |
| MVgreenHblindantiCD20  MV-PNP HblindantiCD20 | human Burkitt’s lymphoma Raji cells  MCL cells (Granta 519)  Primary MCL cells were recovered from surgically  removed spleen tissue or peripheral blood | CD20-targeted measles virus armed with the prodrug convertase purine nucleoside phosphorylase (PNP) that locally converts the active metabolite of F-araAMP to a highly diffusible substance capable of efficiently killing bystander cells | -early prodrug administration controls vector spread, whereas late administration enhances cell killing in vitro and in vivo  -MCL patients derived cells were sensitive to infection  -synergy of F-araAMP with a PNP-armed and CD20-targeted MV with systemic vector inoculation. | (Ungerechts et al., 2007) |
| MV-infected autologous carrier cells (monocytes, endothelial cells or stimulated peripheral blood cells); dual-colour fluorescent MV model | Raji B-cell lymphoma (and ovarian / hepatocellular carcinoma) xenografts | Use MV-infected carrier cells to deliver virus systemically and bypass pre-existing anti-MV neutralising antibodies | -Efficient cell-to-cell transfer via heterofusion, resistant to antibody neutralisation (unlike naked virions)  -Carrier cells delivered MV to tumours after i.v./i.p. injection  -Transferred infection to Raji lymphoma even with neutralising antibodies present  -Repeated carrier injections improved survival; strategy to 'bypass' humoral immunity | (Iankov et al., 2007) |
| MV-eGFP-Pwt: MV-eGFP armed with the P gene from wild-type IC-B strain (encoding IFN-antagonist P/V/C); vs MV-eGFP | BJAB lymphoma, ARH-77 myeloma, activated PBMCs; human myeloma xenografts | Restore MV's natural IFN-evasion (wild-type P gene) to improve intratumoral spread against innate antiviral defences | -MV-eGFP induced IFN in tumour cells and was inhibited by IFN  -MV-eGFP-Pwt showed reduced IFN sensitivity and diminished IFN induction  -i.v. MV-eGFP-Pwt had significantly enhanced oncolytic potency vs MV-eGFP in myeloma xenografts  -OV efficacy improves when natural anti-innate-immunity functions are retained | (Haralambieva et al., 2007) |
| Oncolytic MV-infected CD14+ monocyte/TAM carriers | MM plasmacytomas; KAS6/1 disseminated xenografts | Use tumor-associated-macrophage carriers to deliver MV to plasmacytomas | CD68+ TAM in all 22 patient plasmacytomas; MV-loaded carriers localized to tumors, transferred infection, prolonged survival | (Peng et al., 2009) |
| Recombinant oncolytic MV | Multiple myeloma (modeling) | Mathematical + in vivo modeling of virus–tumor population dynamics | Outcomes from eradication to oscillatory behavior; validated framework to optimize virotherapy | (Dingli et al., 2009) |
| MV-NIS-infected myeloma cell carriers (irradiated MM1) | Disseminated MM (KAS 6/1) in passively immunized mice | BM-tropic carrier cells to deliver MV-NIS despite anti-MV antibodies | Free MV-NIS active only in naive mice; carriers extended survival in naive AND measles-immune mice → bypass humoral immunity | (Liu et al., 2010) |
| MV-PNP H^blind^antiCD20;  MV-GFP H^blind^antiCD20 | -Granta 519 (Mantle cell lymphoma)  -Primary MCL cells | targeted to CD20-expressing cells, blinded to the natural receptors, and armed with the prodrug convertase E. coli purine nucleoside phosphorylase (PNP) | -virus plus fludarabine temporarily control growth of Burkitt (Raji cells) and MCL (Granta cells) lymphoma xenografts in SCID mice  -the targeted virus is strongly specific for CD20-expressing lymphoma cells  -optimized dosing and scheduling for cyclophosphamide, MV-PNP H^blind^antiCD20, and fludarabine, achieved more than tripled survival time. | (Ungerechts et al., 2010) |
| Vaccine MV (Edmonston lineage) | Primary adult B-cell ALL and CLL (patient-derived); ALL xenografts (subcutaneous and disseminated); Bcl-2-overexpressing cell lines | Compare oncolytic potential and kinetics of MV in aggressive ALL vs indolent (Bcl-2-high) CLL using patient material | -ALL formed large multinucleated syncytia with rapid death; CLL showed minimal fusion and slow oncolysis  -Bcl-2 overexpression diminished MV-induced death (mechanism for slow CLL kinetics)  -Intratumoral MV caused complete regression of ALL xenografts and was active in disseminated ALL  -ALL is a highly sensitive MV target | (Patel et al., 2011) |
| Vaccine-related strain MV-NSE | Adult T-cell leukaemia/lymphoma (ATL; HTLV-1-driven CD4+); MET-1/NOD/SCID disseminated xenograft model | Test MV oncolytic therapy against disseminated ATL (vs prior subcutaneous lymphoproliferative models) | -MV-NSE significantly reduced tumour burden  -In high-burden mice, survival increased beyond any other single treatment tested in this model  -Morbidity (cachexia) correlated with IL-6 secretion by MET-1 and host cells, not tumour burden  -MV is promising for generalised lymphoproliferative disease | (Parrula et al., 2011) |
| MV (green)H (blind) antiCD20: fully CD20-retargeted, armed MV; parental vaccine-lineage MV | B-cell NHL patient samples — splenic marginal zone lymphoma (SMZL) and mantle cell lymphoma (MCL); PBMCs/tissue | Quantify spread and specificity of CD20-targeted MV in clinical B-NHL samples and the effect of prior rituximab | -CD20-targeted MV efficiently infected CD20+ SMZL/MCL cells while sparing CD20− cells; parental MV infected both equally  -Prior rituximab (4–8 months before relapse) did not impair infectivity or specificity  -CD20-targeted virotherapy is likely effective even after anti-CD20 therapy | (Yaiw et al., 2011) |
| Vaccine-identical MV armed with the sodium-iodide symporter (MV-NIS) | Mantle cell lymphoma (MCL); primary and metastatic tumour models | Combine MV-NIS radiovirotherapy with systemic 131I and noninvasive imaging; dissect the role of CD46 vs SLAM entry | -In vivo imaging showed homogeneous virus seeding/spread (faster and more intense in metastases)  -MV-NIS + 131I gave more rapid regression than either therapy alone  -Only SLAM-dependent (not CD46) entry sustained efficient spread, regression and prolonged survival  -Refocuses future trials on SLAM-expressing haematologic malignancies | (Miest et al., 2013) |
| Oncolytic MV (vaccine strain) | ATL; HTLV-1+ CD4+ T-cell lines (MT-1/2/4, C8166-45) and HTLV-1− lines (Jurkat, CCRF-CEM); MET-1 and MT-1 in vivo models | Determine why some ATL cells resist MV killing; link resistance to type I IFN (IFNβ) secretion and responsiveness | -All lines were permissive to MV infection, but MT-1 and CCRF-CEM resisted death via IFNβ production  -IFNβ inhibition restored susceptibility to MV-mediated death  -In vivo, IFNβ-producing MT-1 tumours responded poorly while type-I-IFN-deficient MET-1 tumours responded  -Screening tumours for type I IFN could select patients for MV virotherapy | (MC et al., 2014) |
| Attenuated (vaccine) MV | Pediatric B-lineage acute lymphoblastic leukemia (ALL); xenografted and primary patient cells; four B-lineage ALL xenografts in NOD/SCID mice | Test whether MV can eradicate disseminated pediatric ALL ex vivo and in vivo | -MV spread among and killed ALL cells ex vivo while sparing normal blood cells and progenitors  -A few i.v. injections eradicated marrow blasts, controlled CNS disease and gave long-term survival in 3 of 4 xenografts  -Sensitivity did not require high CD150/CD46 or a defective RIG-I/MDA5/IFN pathway  -Dramatically effective against pediatric B-ALL preclinically | (Luhl et al., 2014) |
| MV vaccine strains CAM-70 (non-Edmonston) and Schwarz (Edmonston-derived); CD150-tropic wild-type MV | EBV-transformed B-lymphoblastoid cells (DLBCL model) and EBV+ Burkitt lymphoma with type III latency | Test MV oncolysis of B lymphoma and the role of EBV latency type / CD150 in susceptibility | -Type III (not type I) EBV latency increased susceptibility to MV cytolysis  -EBV latent membrane protein 1 upregulated CD150/SLAM (~3.8-fold), which was critical for killing  -Many lines were more susceptible to CAM-70 than Schwarz (first report of non-Edmonston-strain potential)  -MV OVT is an option for EBV+ DLBCL with type III latency | (Takeda et al., 2014) |
| MV-loaded human bone-marrow-derived mesenchymal stromal cells (BM-MSC carriers) | Precursor B-lineage ALL; systemic/disseminated xenograft model | Use BM-MSCs as carrier cells for systemic MV delivery to overcome anti-MV humoral immunity | -16 adult ALL patients had high-titre anti-MV antibodies  -BM-MSCs were loaded with MV ex vivo and amplified it intracellularly without toxicity  -Live-cell imaging showed viral 'hand-off' from MSC to ALL targets even with antibody present  -Passive anti-MV immunisation abrogated free-MV therapy but NOT MSC-delivered MV — MSCs enable delivery despite immunity | (Castleton et al., 2014) |
| Vaccine-strain measles + mumps (MM) | Wide hematologic range, esp. AML (U937 xenograft; 20 AML patients) | Combine measles+mumps for synergistic oncolysis (± Ara-C) | Synergistic killing via apoptosis; tumor suppression/survival in U937; killed blasts in 16/20 AML patients; enhanced with Ara-C | (Zhang et al., 2014) |
| MV expressing human G-CSF (MVhGCSF) vs backbone MVNSe | B-cell malignancies — Raji (Burkitt) and Nalm-6 (B-ALL) tumour models; CD46-transgenic mice | Test whether arming MV with G-CSF enhances the neutrophil response to MV-infected B-cell tumours | -Neutrophil depletion abrogated MV efficacy in Raji but not Nalm-6  -MVhGCSF enhanced oncolysis in Raji but was detrimental in Nalm-6 (recapitulated by exogenous hGCSF)  -MVhGCSF replicated in CD46-transgenic mice with detectable serum hGCSF and no toxicity  -No 'one-size-fits-all' immune response; each tumour target needs full characterisation | (Dey et al., 2016) |
| MV-CD30  VSC-CD30 | Classical Hodgkin lymphoma (cHL, CD30+); cHL-derived cell lines; cHL xenografts | Selectively destroy CD30+ cHL tumour cells with CD30-retargeted MV and VSV (CD30-specific scFv; in VSV-CD30 the G ORF is replaced by CD30-targeted MV glycoproteins) | -Both viruses were highly CD30-selective (infection blocked by soluble CD30)  -VSV-CD30 gave much higher titres and faster, more efficient killing than MV-CD30  -Intratumoral and systemic VSV-CD30 infected cHL xenografts, slowed growth and prolonged survival  -Supports further preclinical development of VSV-CD30 for cHL and other CD30+ malignancies | (Hanauer et al., 2018) |
| Oncolytic MV (Edmonston) | Multiple myeloma; 37 lines + 23 primary; CD46/p53 | Relate MV sensitivity to p53 status and CD46 | Infection/death tracked CD46, higher in TP53-abnormal cells; p53 activation lowered CD46/infection in p53-WT only → MV rationale for p53-deficient MM | (Lok et al., 2018) |
| MV-CD19/CD3-bsTE | leukemic cell lines REH, Jurkat, K562, and HAL-01,  B-ALL cell line YCUB2  PD-B-ALL: ALL-199 and ALL-265 | oncolytic measles virus (MV-Blina) engineered to locally secrete a bispecific T cell engager (bsTE) targeting  CD19 and CD3 (secBlina) to enhance antitumor immunity  while minimizing systemic toxicity. | superior replication kinetics and cytotoxicity in vitro compared to the parental MV-Edm strain;  infected ALL cells, secreted functional secBlina capable of engaging and activating T cells, leading to selective leukemia cell death;  additive yet heterogeneous anti-leukemic effect, with significant survival benefits and reduced CNS leukemic burden in MV-Blina-treated mice;  not induce either short- or long-term toxicity | (Heinze et al., 2026) |
| **Myxoma virus (MYXV)** | | | | |
| vMyx-GFP  vMyx- M093L-Venus (Fluorescent) | MM cell lines: U266, RPMI-8266, MM.1S and HuNS1;  acute myeloid leukemia cells: HL60 | strong binding to myeloma cells;  and inability to bind or infect CD34+  hematopoietic stem and progenitor cells | MYXV Infects and Kills Human MM Cell Lines In Vitro;  Ex Vivo MYXV Treatment Inhibits In Vivo Systemic Engraftment of MM Cell Lines into NSG Mice;  MYXV Specifically Eliminates MM Cells while Sparing Normal Hematopoietic Stem Cells;  MYXV Kills Human MM Cells by Rapidly Inducing Apoptosis | (Bartee et al., 2012) |
| Myxoma virus | Acute myeloid leukemia (KG-1, THP-1); ex vivo purging; engraftment | Define how MYXV targets AML (binding vs permissiveness) | Prevented marrow engraftment of both lines (MOI≥3); only THP-1 productively infected → binding (not productive infection) sufficient for in vivo purging; heparin blocked it | (Madlambayan et al., 2012) |
| vMyx-GFP  vMyx-RFP | Post-transplant EBV-transformed human lymphomas | Use myxoma virus to prevent EBV-associated malignancies in immunocompromised transplant patients | MYXV showed oncolytic effects on EBV lymphoma cells in vitro and in vivo;  MYXV as prophylactic strategy for preventing post-transplant EBV-transformed  human lymphomas in a highly immunosuppressed mouse xenotransplantation model | (Kim et al., 2015) |
| Myxoma virus | Proteasome-inhibitor-resistant multiple myeloma (Mcl-1/ATF4) | MYXV against bortezomib-relapsed/refractory MM; mechanism | Elimination unaffected by PI resistance; MYXV blocks ATF4 translation after UPR, preventing Mcl-1 upregulation → option for PI-refractory MM | (Dunlap et al., 2015) |
| Myxoma virus (MYXV); construct overexpressing viral decapping enzymes (improved oncolytic version) | Human multiple myeloma cells (clonal plasma-B-cell malignancy) | Define the apoptotic pathway by which MYXV kills myeloma cells (killing occurs even without viral replication) | -MYXV initiates apoptosis via extrinsic initiator caspase-8 → BID cleavage → loss of mitochondrial membrane potential → secondary caspase-9 activation  -Caspase-8 activation is ligand-independent, correlating with depletion of cellular inhibitors of apoptosis (host-protein shutoff)  -A decapping-enzyme-overexpressing MYXV had improved oncolytic potential  -Mechanism unique to oncolytic poxviruses; option for relapsed/refractory myeloma | (Bartee et al., 2016) |
| Autologous donor leukocytes pre-armed with MYXV (BM > PBMC > free virus) | Residual multiple myeloma (BALB/c) | Carrier-cell delivery of MYXV to eliminate MRD | MYXV-armed BM leukocytes superior; eliminated MRD; cured mice immune to MM rechallenge; efficient TME delivery + pro-immune secretion | (Villa et al., 2020) |
| Autologous BM pre-loaded with MYXV (± cyclophosphamide, LCL161, α-PD-1) | Drug-resistant Vk*MYC C57BL/6 myeloma | Ex vivo MYXV-armed autograft alone or with chemo/immunotherapy | BM/MYXV moderate alone; cyclophosphamide delayed onset; synergy with LCL161 and α-PD-1 controlled established MM, improved survival; survivors immune | (Villa et al., 2022) |
| Oncolytic myxoma virus | Multiple myeloma (human U-266, murine MOPC-315) | Investigate MYXV effect on autophagy | ↑autophagy markers (ATG-5/Beclin-1 at 24 h; LC3B at 48 h); autophagosomes by TEM → antimyeloma effect via autophagy | (Yeşilaltay et al., 2024) |
| Myxoma virus + lenalidomide + bortezomib | Multiple myeloma (lines and primary) | MYXV combination with standard MM drugs; entry receptor | Reduced viability, ↑caspase-9/apoptosis; only CD86 blockade reduced entry; higher early apoptosis (newly diagnosed and refractory) → patient-tailored MYXV combinations | (Yeşilaltay et al., 2024) |
| **Newcastle disease virus (NDV)** | | | | |
| NDV strains: mesogenic Roakin (active and UV-inactivated) and lentogenic B1 (attenuated) | Burkitt lymphoma Daudi cells | Investigate the destructive effect of NDV strains on Burkitt lymphoma cells | -High-multiplicity infection (active or UV-inactivated mesogenic, and egg-grown lentogenic B1) inhibited DNA synthesis, arrested multiplication and killed cells  -B1 grown in chicken fibroblasts was only moderately active  -Cytolysis linked to increased membrane permeability (raised 51Cr release) from massive viral adsorption/penetration | (Tzadok-David et al., 1995) |
| NDV (influenza A/PR/8/34 as comparator) | Lymphoma cells (Daudi, HD-Mar) vs lymphoblastoid (Milstein) and resting/mitogen-activated peripheral blood lymphocytes (PBL) | Compare NDV susceptibility of malignant lymphoma cells vs normal/non-malignant lymphocytes | -Lymphoma cells more sensitive than lymphoblastoid/resting PBL; mitogen-activated PBL reached lymphoma-like sensitivity  -Daudi cells mixed with PBL were more sensitive (70% vs 30% mortality)  -Sensitivity was unrelated to adsorption efficiency (similar across lines); influenza adsorbed but was non-cytotoxic  -NDV preferentially damages lymphoma vs normal cells | (Bar-Eli et al., 1996) |
| NDV strains AF2240 (velogenic), V4-UPM (lentogenic) | WEHI-3B murine leukemia | Determine oncolytic/apoptotic potential on leukemia | Both cytotoxic (CD50 2 and 8 HAU); inhibited proliferation; apoptosis (↑caspase-3, DNA laddering, sub-G1) | (Alabsi et al., 2011) |
| NDV-MLS, a low virulence attenuated lentogenic strain | human large B-cell lymphoma cell line (SU-DHL-4),  -primary canine-derived B-cell lymphoma cells | In vitro and in vivo in dog with T-cell lymphoma | -Malignant Lymphocytes of Human and Canine Origin Are Selectively Killed by the NDV-MLS  -Peripheral Blood Mononuclear Cells Are Less Sensitive to the NDV-MLS  -Human Non-Malignant B-Cells Are Not Sensitive to the Cytotoxic Effect of the NDV-MLS | (Sanchez et al., 2015) |
| NDV (Iraqi strain), combined with rituximab or doxorubicin | Plasmacytoma cells and non-Hodgkin lymphoma cells | Newcastle disease virus, rituximab, and doxorubicin combination | -NDV synergised with low-dose doxorubicin on plasmacytoma (p53-independent) and on NHL cells (p53-dependent)  -NDV + rituximab showed enhanced, p53-independent cytotoxicity  -Proposes a novel combination modality for haematological malignancies | (Al-Shammari et al., 2016) |
| Attenuated Iraqi NDV strain AMHA1 | NHL (SR), multiple myeloma (COLO 677) and monocytic leukemia (THP-1) cell lines | Evaluate AMHA1 NDV induction of apoptosis via caspase-dependent and -independent pathways | -Dose-dependent cytolysis across all three lines (MTT); significant apoptosis induction (AO/PI, P<0.0001)  -Increased caspase-8 and -9 (dependent pathway) plus apoptosis-inducing factor and endonuclease G (independent pathway)  -AMHA1 NDV kills haematological tumour cells via both apoptotic routes; strong antitumor potential | (Mohammed et al., 2019) |
| NDV strain AF2240 (velogenic viscerotropic) | WEHI-3B myelomonocytic leukemia | Ultrastructural characterization of NDV-induced apoptosis | SEM/TEM showed altered morphology, reduced viability; early apoptosis 6 h post-inoculation | (Ali-Saeed et al., 2024) |
| NDV-GFP | Feline lymphoma cells isolated from a FeLV-positive patient with thymic lymphoma | Explore the oncolytic potential of NDV expressing green fluorescent protein (NDV-GFP) in feline lymphoma cells, as an alternative therapy for cats that relapse or are unresponsive to conventional chemotherapy; assess viral infection, replication and apoptosis induction in vitro. | The NDV-GFP infected, replicated, and induced apoptosis in feline lymphoma cells. | (Alves et al., 2025) |
| **Bovine pestivirus** |  |  |  |  |
| Bovine viral diarrhea virus (BVDV) — a non-human pestivirus that interacts with CD46 | Human multiple myeloma cell lines; CD138+ plasma cells from patient bone marrow; MM xenografts (NOD-SCID) | Test a non-human oncolytic virus (BVDV) to bypass anti-viral immunity; assess CD46, BVDV cytotoxicity and bortezomib combination | -Human myeloma cells were selectively sensitive to BVDV (increased apoptotic markers)  -Patient bone marrow showed a selective decrease of viable CD138+ cells  -Bortezomib pre-treatment synergistically increased BVDV cytotoxicity  -In vivo BVDV reduced tumour burden; first evidence of a direct oncolytic effect of BVDV in myeloma | (Marchica et al., 2020) |
| **Reovirus** | | | | |
| Reovirus (unattenuated, wild-type), used ex vivo | Monocytic and myeloma cell lines; ex vivo lymphoma, myeloma and Waldenström macroglobulinemia patient cells; apheresis/CD34+ grafts | Test reovirus as an ex vivo purging agent to remove contaminating tumour cells from autologous stem-cell grafts | -Reovirus significantly reduced viability of cell lines and ex vivo DLBCL, CLL, Waldenström and small lymphocytic lymphoma cells  -Complete purging of apheresis admixtures for many tumours (incomplete for myeloma, Burkitt, follicular)  -Did not harm CD34+ stem cells or their colony-forming capacity  -Attractive ex vivo purging strategy for autologous stem-cell transplantation | (Thirukkumaran et al., 2003) |
| Reovirus (wild-type) | Burkitt lymphoma Raji cells; persistently infected (PI) and 'cured' Raji; SCID-mouse xenografts | Address the fate and risk of reovirus-resistant tumour cells emerging after oncolysis | -PI and 'cured' Raji resisted reovirus in vitro  -PI cells were non-tumorigenic in SCID mice; 'cured' cells regained tumorigenicity  -'Cured'-cell xenografts were highly susceptible to reovirus oncolysis in vivo (tumour proteolytic environment facilitates infection)  -PI/cured states are unlikely to be major barriers to reovirus therapy | (Alain et al., 2006) |
| reovirus serotype 3 | mantle cell lymphomas cell lines (Z138, HBL2, JVM-2);  FL cell line Dohh2 | Assess reovirus mediated oncolysis in a panel of human MCL cell lines | cytopathic effect of reovirus infection ranged from high to very limited and correlated with levels of Ras activation;  a single reovirus injection intra-tumorally resulted in complete regression of both the injected and the contra-lateral tumor in a subcutaneous bi-tumor model | (Alain et al., 2010) |
| Reovirus | Acute myeloid leukemia (lines, primary); NK/PBMC | Direct cytotoxicity + NK-mediated innate immunity | Replicated in and killed AML, reduced primary viability; induced IFNα/RANTES; activated NK (degranulation, IFNγ) enhancing AML killing | (Hall et al., 2012) |
| Reovirus | Multiple myeloma (7 lines, 7 patient specimens; NOD/SCID); HSCs | Test MM targeting while sparing hematopoietic stem cells | 6/7 lines, 5/7 specimens sensitive; reduced tumor weight/marrow infiltration; HSC repopulation unaffected → systemic MM candidate | (Thirukkumaran et al., 2012) |
| Reolysin (reovirus) + bortezomib | Multiple myeloma (xenograft + syngeneic bone-disease) | Reovirus + proteasome inhibitor; ER-stress/NOXA mechanism | Induced ER stress and NOXA-dependent apoptosis; bortezomib amplified ER stress/NOXA/apoptosis and decreased tumor burden in vivo | (Kelly et al., 2012) |
| Reovirus (Reolysin) | Multiple myeloma | Characterize cell-death mechanism | In addition to apoptosis, reovirus upregulates autophagy during MM oncolysis (first demonstration) | (Thirukkumaran et al., 2013) |
| Reovirus (live vs dead) | Multiple myeloma (RPMI 8226, MM1S, H929, U266); apheresis purging; NOD/SCID | Ex vivo autograft-purging agent for MM | Significant purging; complete ex vivo purge of RPMI 8226 (no relapse); 100% survival of LV-purged-autograft recipients; HSCs spared | (Thirukkumaran et al., 2014) |
| Reolysin (reovirus); JAM-A | Multiple myeloma (primary, most lines; bortezomib-resistant) | Identify JAM-A (not RAS) as killing determinant biomarker | JAM-A required for Reolysin death; ↑ at relapse and in bortezomib-resistant cells (more sensitive) → predictive biomarker for r/r MM | (Kelly et al., 2015) |
| Reovirus type 3 dearing strain (Reolysin | chronic lymphocytic  leukaemia | as a direct cytotoxic agent and as an immunomodulator | directly cytotoxic against CLL, which requires replication-competent virus;  phenotypically and functionally activates patient NK cells via a monocyte-derived interferon-α (IFNα)-dependent mechanism;  enhances ADCC-mediated killing of CLL in combination with anti-CD20 antibodies | (Parrish et al., 2015) |
| Reovirus (Reolysin / pelareorep; wild-type oncolytic reovirus) | Ten canine lymphoma cell lines; canine lymphoma subcutaneous tumours in NOD/SCID mice | Test reovirus susceptibility of canine lymphoma (extending reovirus veterinary oncology beyond mast cell tumour) | -Reovirus-induced death, replication and infectivity in 4 of 10 lines with variable susceptibility  -Ras activation did not correlate with susceptibility  -Susceptible lines died by apoptosis (Annexin V/PI, PARP cleavage, caspase-dependent)  -A single intratumoral injection suppressed canine lymphoma xenograft growth  -Canine lymphoma is less susceptible than canine mast cell tumour | (Hwang et al., 2016) |
| Reolysin + HDAC inhibitors | Multiple myeloma (in vitro/in vivo) | HDACi to upregulate JAM-A and potentiate reovirus | JAM-A epigenetically regulated; HDACi increased JAM-A/acetylation and potentiated reovirus killing in vitro/in vivo | (Stiff et al., 2016) |
| Reovirus type 3 (Reo3) | Leukemia (HL60) | Dissect apoptosis mechanism (PKR pathway) | Inhibited viability and induced apoptosis via PKR activation (↑PKR/p-PKR, Bax, cleaved caspase-3; ↓Bcl-2); 2-AP reduced apoptosis | (Li et al., 2019) |
| Oncolytic reovirus | Multiple myeloma in BM niche (5TGM1-Kalw/RijHSD; human co-cultures) | Characterize reovirus-induced cell-mediated immunity in resistant niche | Reduced burden/bone disease; activated NK and effector-memory CD8+ (correlating with response); stroma-protected MM killed by reovirus-activated NK/CTLs | (Muller et al., 2021) |
| Clinical oncolytic reovirus (Reolysin / Pelareorep) ± JAK inhibitor ruxolitinib | T-cell lymphoma (TCL); HTLV-1-positive vs -negative TCL cells | Determine why HTLV-1+ TCL resists reovirus and whether JAK/STAT inhibition overcomes it | -HTLV-1− TCL was highly sensitive to reovirus; HTLV-1+ cells were resistant with poor viral replication  -HTLV-1 infection conferred resistance and upregulated IL-13/STAT5  -Ruxolitinib suppressed phospho-STAT5 and antiviral MX1 and enhanced reovirus anti-TCL activity in both HTLV-1+ and − cells  -JAK/STAT inhibition overcomes reovirus resistance | (Islam et al., 2021) |
| Reovirus + NAMPT inhibitor FK866 (NAD+ depletion) | Multiple myeloma (12 lines; KMS12; patient CD138+; in vivo) | Metabolic sensitization (deplete NAD+) to reovirus | NAD+ inversely correlated with oncolysis; FK866 sensitized MM (esp. p53-WT); mitochondrial dysfunction → autophagic death; greater antitumor effect in vivo | (Kennedy et al., 2022) |
| Oncolytic reovirus via UC-MSC-derived exosomes | Acute myeloid leukemia | Cell-free exosome carrier (evade neutralizing antibodies) | UC-MSCs transferred reovirus to AML via exosomes (clathrin-mediated endocytosis/macropinocytosis); exosomes exerted anti-AML lysis | (Yang et al., 2024) |
| Reovirus + SMAC/BH3-mimetic apoptotic modulators | Acute myeloid leukemia (lines, patients, healthy PBMC) | Combine reovirus cytokine killing with apoptotic modulators | OV-stimulated PBMC cytokines (IFNα, TNFα) killed AML; mimetics enhanced bystander and direct-infection death (live and UV-inactivated virus) | (Askar et al., 2026) |
| **Sindbis viral vectors** | | | | |
| Sindbis viral vectors | Mouse BW5147 malignant T-cells; human lymphoma/leukemia lines (Jurkat, Karpas, CEM, DHL, JB); transgenic CD4-Rgr thymic lymphoma; in vivo (C3H×AKR)F1 model | Establish efficacy of Sindbis vectors against blood cancers and define tropism (laminin receptor, LAMR, and other factors) | -Efficient apoptosis in mouse BW5147 cells but only low-level infection of human lymphoma/leukemia lines  -LAMR expression alone did not predict infectivity (additional tropism factors involved)  -In vivo, Sindbis targeted BW5147 tumours and significantly prolonged survival; less efficient in transgenic thymic lymphoma  -Potential against haematopoietic malignancies; human susceptibility needs further study | (Suzme et al., 2012) |
| Sindbis virus (SV) vectors combined with agonistic anti-4-1BB (CD137) monoclonal antibody | A20 non-Hodgkin B-cell lymphoma (luciferase-tagged) in BALB/c mice | Combine SV vectors with α4-1BB costimulatory mAb as cancer immunotherapy; dissect mediating molecular/metabolic pathways | -SV + α4-1BB completely eradicated A20 lymphoma (neither monotherapy did)  -Synergy boosted T-cell cytotoxicity, IFNγ, proliferation, migration and glycolysis  -All survivors developed long-lasting antitumor immunity (rejected A20 rechallenge)  -Identifies cytokine/chemokine/metabolic pathways supporting translation of SV + agonistic mAb | (Yu et al., 2019) |
| **Oncolytic vaccinia virus (OVV)** | | | | |
| VV with mutations in the viral thymidine kinase and viral growth factor genes + luciferase or GFP | -Lymphoma cell lines 6780, 6814, and 8946  -primarily murine B-cell lymphomas | CIK-vvDD: tumor-targeting immune cell line [cytokine-induced killer (CIK) cells as carrier for OVV  combine effective cellular delivery with synergistic tumor killing | -CIK-vvDD traffick to and target stem cell–like residual cancer cells  - prevented relapse by creating a long-term, disease-free state, with acquired immunity to the tumor  -Can be applied before or after conventional therapy  Showing restoring immune surveillance as critical for maintenance of a disease-free state | (Contag et al., 2010) |
| VV-miR-34a;  VV-Smac | multiple myeloma (MM) cell lines and xenograft model | OVV (TK deletion) expressing anti-tumor genes, miR-34a and Smac  VV-miR-34a combined with VV-Smac | -Replicate in and kill human MM cells in vitro and in vivo  -Combination of VV-miR-34a and VV-Smac synergistically improved anti-tumoral efficacy | (Lei et al., 2016) |
| TK-deleted, let-7a-miRNA-regulated vaccinia (doubly regulated) | Multiple myeloma; SCID xenograft | TK deletion + miRNA detargeting for myeloma-specific safety | Myeloma highly susceptible; unregulated VV toxic/lethal, but regulated virus stayed localized, regressed tumor and improved survival | (Futami et al., 2017) |
| OVV-ING4 | Human lymphoma cell lines (Raji and SU-DHL-4)  murine lymphoma cell line (EL4 and A20) | Combination of an adjuvant of embelin with OVV (1) increase the OVV replication by mitigating antiviral innate immunity; (2) suppresses constitutive STAT3 phosphorylation  and mitigates OVV-induced activation of STAT3 in lymphoma | Embelin Enhanced OVV-Mediated Lymphoma Cell Lysis  Embelin Enhances OVV-Mediated Antitumor Immunity in a Xenograft Model of Lymphoma | (Wang et al., 2020) |
| OVV-BECN1 (oncolytic vaccinia expressing Beclin-1) | Leukemia and multiple myeloma lines (incl. caspase-3 shRNA); xenografts | Arm vaccinia with Beclin-1 for autophagic, apoptosis-independent death | Higher infectivity in leukemia; apoptosis-independent autophagic death superior to parent OVV; SIRT-1↑→LC3 deacetylation; efficacy in vitro/in vivo | (Lei et al., 2020) |
| OVV expressing manganese superoxide dismutase (OVV-MnSOD) | Poorly immunogenic / minimally inflammatory tumours in immunocompetent mice (general solid-tumour models; not lymphoma-specific) | Use OVV-MnSOD to improve lymphocyte trafficking into tumours and enhance anti-PD-L1 checkpoint therapy | -Intratumoral OVV-MnSOD induced intratumoral inflammation and marked tumour regression  -OVV-MnSOD + anti-PD-L1 improved outcomes where each monotherapy was ineffective  -Supports OV-driven TME remodeling to sensitise 'cold' tumours to checkpoint blockade | (Lou et al., 2021) |
| OVV‑Beclin1 | patients with NHL  OCI‑LY3 and Pfeiffer cells | Increase intratumoral Beclin1 expression levels as a means of inducing autophagic cell death | -OVV‑Beclin1 induces autophagic death of NHL cells  -OVV‑Beclin1 enhances the therapeutic efficacy of R‑CHOP against lymphoma in vitro and in vivo | (Xie et al., 2021) |
| OVV-CD19BiTE | lymphoma cells (Raji, Pffeifer, Farage, and K562/CD19) | -OVV encoding bispecific T-cell engagers  - immunomodulatory effects of oncolysis in combination with local BiTE-mediated T-cell recruitment and T-cell activation | -OVV-CD19BiTE-infection induce activation and proliferation of human T cells in vitro  - OVV-CD19BiTE-infection recruit an immune response to a tumor and stimulating T-cells proliferation in vivo  - long-term tumor remissions and prolonged survival in tumor xenograft mouse | (Lei et al., 2022) |
| OVV-RFP | A20 (murine lymphoma cells) | SFOVV@IR1061-combinination therapy:  -oncolytic vaccinia virus (OVV)  -the second near-infrared (NIR-II) fluorescent dye IR1061  -ncapsulated within Sophora flavescens-derived extracellular vesicles (SFNPs) | - enhanced tumor targeting and in vivo stability,  - enables high-resolution PAI for precise tumor visualization and therapeutic monitoring.  - effectively induces ICD, activates both innate and adaptive immunity, and improves tumor-targeting efficiency while minimizing systemic toxicity | (Ji et al., 2025) |
| OVV-mCD47nb;  OVV-hCD47nb-G1 | -A20 cells (BALB/c B cell lymphoma line)  -Raji cells | -Direct oncolysis of tumor cells;  -Fc-mediated enhancement of innate immune cell cytotoxicity;  -Recruitment and activation of both innate and adaptive immune cells;  -Immune checkpoint modulation in macrophages | significant tumor regression and prolonged survival | (Li et al., 2025) |
| OVV-hIL18 | A20, Raji, and  Jeko-1 cells | IL-18 reversed OVV-induced immunosuppression by promoting M1 polarization and reducing Tregs, boosting  OVV-mediated immunity | OVV-hIL18 had enhanced in vitro oncolysis, significantly inhibited tumor growth, prolonged survival in animal models without overt toxicity, and increased CD4⁺/CD8⁺ T cell infiltration, effector cytokine production and relieved T cell exhaustion in TME. | (Wu et al., 2026) |
| OVV-αCD47nb (oncolytic vaccinia encoding anti-CD47 nanobody) | Multiple myeloma (murine models) | Direct oncolysis + CD47–SIRPα blockade; ± bortezomib | Enhanced macrophage phagocytosis; suppressed growth, extended survival, no hematologic toxicity; M1 polarization + CD8 infiltration; synergy with bortezomib | (Pan et al., 2026) |
| **Vesicular stomatitis virus (VSV)** | | | | |
| Recombinant VSV expressing the E. coli cytosine deaminase/uracil phosphoribosyltransferase fusion suicide gene (VSV-C:U) | Syngeneic A20 B-cell lymphoma (and TSA mammary carcinoma) in BALB/c mice | Arm VSV with CD/UPRT to convert 5-fluorocytosine (5-FC) to 5-FU and enhance oncolysis via a bystander effect | -VSV-C:U generated active CD/UPRT, converting 5-FC to 5-FU with a strong bystander effect  -Intratumoral VSV-C:U + systemic 5-FC significantly reduced A20 and TSA tumour growth vs controls  -Marked activation of IFN-γ-secreting cytotoxic T cells  -Suicide gene potentiates VSV oncolysis without altering oncolytic kinetics | (Porosnicu et al., 2003) |
| wtVSV;  rVSV-GFP | primary patient leukemia  HTLV-1 T-cell lines: MT-2 and MT-4  EBV transformed B-cell chronic lymphocytic leukemia (B-CLL) I-83;  EBV-negative B-CLL WSU | VSV targets genetic defects unique to tumor cells to facilitate its replication | Ex vivo primary ATL cells were permissive for VSV and underwent rapid oncolysis;  No viral replication and oncolysis in HTLV-1-infected, nonleukemic cells from patients with HTLV-1-associated myelopathy/tropical spastic paraparesis (HAM/TSP), and in naive CD4+ T-lymphocytes from normal individuals or in ex vivo cell samples from patientswith chronic lymphocytic leukemia (CLL) | (Cesaire et al., 2006) |
| VSV(Δ51)-NIS (M51-deleted, encodes NIS) | Multiple myeloma lines/primary; 5TGM1 (s.c. and orthotopic) | Attenuated VSV-NIS radiovirotherapy (+131I) and imaging | Specific oncolysis, high replication, regression, no neurotoxicity; VSV(Δ51)-NIS + 131I enhanced regression and survival in immunocompetent mice | (Goel et al., 2007) |
| VSV + small-molecule BCL-2 antagonist EM20-25 | Primary ex vivo CLL cells (CD5+CD19+); Karpas-422 and Granta-519 B-lymphoma lines; healthy-donor PBMCs | Overcome CLL resistance to VSV oncolysis by inhibiting anti-apoptotic BCL-2 | -VSV + EM20-25 synergised in primary CLL (combination index 0.5; P<0.0001), with a 19-fold therapeutic index vs healthy PBMCs  -Increased apoptosis in B-lymphoma lines via the intrinsic mitochondrial pathway  -EM20-25 blocked BCL-2–BAX dimerisation, sensitising cells to VSV  -BCL-2 inhibitors may improve VSV oncolysis in apoptosis-defective malignancies | (Tumilasci et al., 2008) |
| VSV (oncolytic; disrupts de novo protein synthesis) | Tumour cells overexpressing Mcl-1 (chemoresistant); tumour mouse model (+ doxorubicin) | Exploit VSV shutdown of protein synthesis to deplete short-lived Mcl-1 and overcome chemoresistance | -VSV infection rapidly reduced Mcl-1 by proteasomal degradation while other Bcl-2 members were unchanged  -Mcl-1 rescue inhibited apoptosis, confirming its role in VSV-induced death  -VSV + chemotherapy was synergistic in vitro (reversed by Mcl-1 rescue or Bax/Bak knockdown)  -VSV + doxorubicin was superior to either alone in vivo; VSV overcomes chemoresistance by eliminating Mcl-1 | (Schache et al., 2009) |
| VSV + BCL-2 inhibitor obatoclax (GX15-070) | Primary CLL; A20 lymphoma (SCID) | Overcome BCL-2 resistance to VSV oncolysis | Synergistic death in primary CLL; reduced A20 growth; mitochondrial apoptosis (caspase-3/9, cytochrome c, BAX); NOXA a key inducer | (Samuel et al., 2010) |
| VSV; combined with recombinant Flt3 ligand (rFlt3L) | VSV-sensitive E.G7 T lymphoma and VSV-resistant B16 melanoma models | Test whether boosting tumour-associated dendritic cells (rFlt3L) plus VSV improves antitumor immunity and antigen presentation | -VSV + rFlt3L improved survival in both models but independently of an adaptive CD8 T-cell response  -VSV actively infected tumour-associated DC, reducing their viability and blocking migration to draining lymph nodes  -VSV interferes with DC function and abrogates tumour antigen presentation (a caution for immune priming) | (Leveille et al., 2011a) |
| VSV-MΔ51 expressing the cytosine deaminase/uracil phosphoribosyltransferase (CD::UPRT) suicide gene + 5-fluorocytosine prodrug | VSV-sensitive/-resistant panel — B-lymphoma Karpas, prostate PC3, breast MCF7/TSA, melanoma B16-F10 | Combine VSV-CD::UPRT with the 5-FC prodrug to enhance bystander oncolysis; optimise dosing and timing | -Combination increased killing of non-infected bystander cells via 5-FC toxic derivatives  -Synergy of VSV-MΔ51 with the active drug 5-fluorouracil  -Optimising VSV replication timing and 5-FC bioavailability improved survival of TSA-bearing mice  -Highlights the need to optimise each step of multicomponent OV therapy | (Leveille et al., 2011b) |
| VSV-GFP;  VSV-mIFNβ-NIS; VSV-hIFNβ-NIS | -Murine myeloma cells (5TGM1 and MPC-11)  -Murine melanoma cells (B-16) | -express interferon-β (IFNβ) to confer myeloma specificity and to reduce neurotoxic potential  -thyroidal sodium iodide symporter (NIS) to permit noninvasive in vivo monitoring of virus spread; as potency enhancement by combination with ^131^I radiotherapy | a single-shot intravenous therapy with VSV-IFNβ-NIS to cure advanced syngeneic myelomas in immunocompetent mice | (Naik et al., 2012) |
| VSV + triptolide (natural anticancer compound; type I IFN-signalling inhibitor) | OV-resistant cancer cells (e.g. prostate PC3, TSA mammary); in vitro and in vivo xenografts (not lymphoma-specific) | Use triptolide to block type I IFN signalling and sensitise OV-resistant cells to VSV | -Triptolide increased VSV replication in resistant cells dose- and time-dependently by blocking type I IFN signalling downstream of IRF3  -Enhanced VSV-induced apoptosis (annexin-V, cleaved caspase-3)  -VSV + triptolide delayed tumour growth and prolonged survival in vivo  -IFN inhibition sensitises resistant cells to VSV oncolysis | (Ben Yebdri et al., 2013) |
| VSV | ex vivo CLL patient samples. | BCL-2 inhibitors, obatoclax, and ABT-737 | -VSV + obatoclax or ABT-737 had a combined therapeutic effect in CLL cells from untreated and standard-of-care-refractory patients  -Combination induced both apoptosis and autophagy (LC3-II accumulation, p62 degradation); blocking early autophagy (3-MA) increased apoptosis  -BCL-2 inhibitors disrupted Beclin-1–BCL-2/MCL-1 interactions, biasing cells toward autophagy and apoptosis  -Couples metabolic change with BCL-2 inhibition to drive VSV cytotoxicity | (Samuel et al., 2013) |
| VSV-IFNβ (attenuated) | 5TGM1 myeloma with CNS/meningeal deposits | Cautionary therapeutic-index study with CNS tumor seeding | Potent vs s.c. tumors without neurotoxicity, but i.v.-seeded meningeal deposits → fatal meningoencephalitis on VSV infection → caution with CNS deposits | (Yarde et al., 2013) |
| Oncolytic VSV + bortezomib | Multiple myeloma (in vitro; two syngeneic models) | VSV + proteasome inhibitor combination | Antagonistic in vitro (bortezomib blocks VSV NF-κB-dependent spread) but additive in vivo (greater tumor reduction than VSV alone) | (Yarde et al., 2013) |
| Non-replicating rhabdovirus-derived particles (NRRPs; VSV-based) | Acute leukemia (lines, blast-crisis models, MDR-AML patients) | High-dose non-replicating particles to bypass live-virus safety/spread limits | Multimodal cytolysis + antitumor immunity; normal cells protected by IFN; eradicated disseminated leukemia (first non-replicating virus-derived agent) | (Batenchuk et al., 2013) |
| VSV-gp160G, | HTLV-1-associated adult T-cell leukemia/lymphoma (ATL; CD4/CD25+); patient-derived ATL in NSG mice; primary CD4+ T cells as control | Retarget VSV to CD4+ transformed cells (G replaced by an HIV-1 gp160/VSV-G hybrid fusion protein) to gain ATL selectivity and eliminate neurotoxicity | -VSV-gp160G targeted only CD4+ cells and retained robust oncolytic activity against ATL  -Highly attenuated in / non-toxic to primary CD4+ T cells; no neurotoxicity even in NSG mice  -Potent oncolysis and significant survival benefit in patient-derived ATL xenografts  -Promising CD4-targeted VSV for ATL, alone or with established therapies | (Betancourt et al., 2015) |
| VSV-mIFNβ-NIS;  VSV-GFP | -Murine AML cell line C1498  -Mononuclear cells (MNCs) from peripheral blood and bone marrow of AML patients | VSV-IFNb-NIS combine with anti-PD-L1 immunotherapy | -IV therapy with oncolytic VSV-IFNb-NIS virus extends  survival of immunocompetent mice with AML  -The therapeutic outcome of VSV-IFNb-NIS–treated mice  with AML is augmented by anti-PD-L1 immunotherapy  - Primary AML cells from clinical samples were susceptible to  infection and killing by VSV | (Shen et al., 2016) |
| (VSV)-IFNβ-NIS | Pet dogs with spontaneous cancers (9 dogs), including two with high-grade peripheral T-cell lymphoma | Comparative-oncology study of a single intravenous dose of VSV-IFNβ-NIS to assess tolerability, shedding, pharmacokinetics and efficacy and inform human translation | -Single i.v. dose was well tolerated and safe; no infectious virus shedding  -Two peripheral T-cell lymphoma dogs had rapid but transient remission with transient, self-resolving hepatotoxicity  -Elevated blood VSV RNA correlated with disease remission  -Informed dose and indication selection for clinical translation of i.v. VSV-IFNβ-NIS | (Naik et al., 2018) |
| VSVΔG encoding HTLV-1 receptors (GLUT1/NRP1/HSPG; VSVΔG-GL/-NP/-GLN/-GLNS) | HTLV-1 Env+ infected cells (ATL prevention); humanized mice | Retarget VSV to HTLV-1 Env+ cells to prevent ATL | VSVΔG-GL/-NP eliminated infected cells in vitro; VSVΔG-NP prevented HTLV-1 leukocytosis and cleared Env+ cells in vivo → prophylactic anti-HTLV-1 virotherapy | (Tezuka et al., 2018) |
| **Other oncolytic viruses (rotavirus, Mengovirus, Pteropine orthoreovirus)** |  |  |  |  |
| Tumor-cell-adapted rotavirus isolate Wt1-5 | Human acute lymphoblastic leukemia cell line Reh | Identify the surface receptors and cell-death markers of rotavirus infection in ALL cells | -Wt1-5 used HSPs (90/70/60/40, Hsc70), PDI and integrin beta3 for entry  -Induced membrane-permeability change, mitochondrial dysfunction, DNA fragmentation and apoptosis  -Candidate oncolytic agent for lymphoblastic leukemia | (Guerrero et al., 2020) |
| Tumor-cell-adapted rotaviruses (Wt1-5, WWM, TRUYO, ECwt-O, WTEW) | Murine myeloma Sp2/0-Ag14 | Assess rotavirus oncolytic potential in myeloma | Infected and lysed Sp2/0 (HSP/PDI/integrin-β3 entry); death via membrane change, chromatin condensation, DNA fragmentation (apoptosis) → candidate OVs | (Guerrero et al., 2020) |
| microRNA-detargeted oncolytic Mengovirus (polyC-truncated; miR-124/133b/208a targets; MC0-NC lead) | MPC-11 murine plasmacytoma/MM | Optimize Mengovirus therapeutic index (polyC truncation + miRNA detargeting) | polyC-deleted MC0-NC gave faster responses and 75% durable complete responses vs polyC-reconstituted; retained miRNA targets (safety) → lead candidate | (Penza et al., 2023) |
| Sikamat virus (PRV7S; Pteropine orthoreovirus) | AML (THP-1), CML (K562); AML-M5 cancer stem cells | Evaluate novel orthoreovirus OV for myeloid leukemia incl. CSCs | Infected/replicated, reduced viability via caspase apoptosis (± necroptosis); efficacy vs CSCs; well tolerated in mice, no persistence | (Siew et al., 2025) |

Al-Shammari, A.M., Rameez, H., and Al-Taee, M.F. (2016). Newcastle disease virus, rituximab, and doxorubicin combination as anti-hematological malignancy therapy. *Oncolytic Virother* 5**,** 27-34. doi: 10.2147/OV.S95250.

Alain, T., Kim, M., Johnston, R.N., Urbanski, S., Kossakowska, A.E., Forsyth, P.A., et al. (2006). The oncolytic effect in vivo of reovirus on tumour cells that have survived reovirus cell killing in vitro. *Br J Cancer* 95(8)**,** 1020-1027. doi: 10.1038/sj.bjc.6603363.

Alain, T., Muzik, H., Otsuka, S., Chan, S., Magliocco, T., Diaz, R., et al. (2010). Susceptibility of mantle cell lymphomas to reovirus oncolysis. *Leuk Res* 34(1)**,** 100-108. doi: 10.1016/j.leukres.2009.05.020.

Alves, T.G.L., Xavier, P.L.P., Garnica, T.K., Rochetti, A.L., Qazi, T.J., Vendramini, T.H.A., et al. (2025). Oncolytic potential of Newcastle Disease Virus in feline lymphoma cells: an in vitro evaluation. *Front Vet Sci* 12**,** 1484947. doi: 10.3389/fvets.2025.1484947.

Angelova, A., Barf, M., Just, A., Leuchs, B., Rommelaere, J., and Ungerechts, G. (2024). H-1 Parvovirus-Induced Oncolysis and Tumor Microenvironment Immune Modulation in a Novel Heterotypic Spheroid Model of Cutaneous T-Cell Lymphoma. *Cancers (Basel)* 16(15). doi: 10.3390/cancers16152711.

Angelova, A.L., Aprahamian, M., Balboni, G., Delecluse, H.J., Feederle, R., Kiprianova, I., et al. (2009). Oncolytic rat parvovirus H-1PV, a candidate for the treatment of human lymphoma: In vitro and in vivo studies. *Mol Ther* 17(7)**,** 1164-1172. doi: 10.1038/mt.2009.78.

Au, G.G., Lincz, L.F., Enno, A., and Shafren, D.R. (2007). Oncolytic Coxsackievirus A21 as a novel therapy for multiple myeloma. *Br J Haematol* 137(2)**,** 133-141. doi: 10.1111/j.1365-2141.2007.06550.x.

Bar-Eli, N., Giloh, H., Schlesinger, M., and Zakay-Rones, Z. (1996). Preferential cytotoxic effect of Newcastle disease virus on lymphoma cells. *J Cancer Res Clin Oncol* 122(7)**,** 409-415. doi: 10.1007/BF01212880.

Bartee, E., Chan, W.M., Moreb, J.S., Cogle, C.R., and McFadden, G. (2012). Selective purging of human multiple myeloma cells from autologous stem cell transplantation grafts using oncolytic myxoma virus. *Biol Blood Marrow Transplant* 18(10)**,** 1540-1551. doi: 10.1016/j.bbmt.2012.04.004.

Bartee, M.Y., Dunlap, K.M., and Bartee, E. (2016). Myxoma Virus Induces Ligand Independent Extrinsic Apoptosis in Human Myeloma Cells. *Clin Lymphoma Myeloma Leuk* 16(4)**,** 203-212. doi: 10.1016/j.clml.2015.12.005.

Ben Yebdri, F., Van Grevenynghe, J., Tang, V.A., Goulet, M.L., Wu, J.H., Stojdl, D.F., et al. (2013). Triptolide-mediated inhibition of interferon signaling enhances vesicular stomatitis virus-based oncolysis. *Mol Ther* 21(11)**,** 2043-2053. doi: 10.1038/mt.2013.187.

Betancourt, D., Ramos, J.C., and Barber, G.N. (2015). Retargeting Oncolytic Vesicular Stomatitis Virus to Human T-Cell Lymphotropic Virus Type 1-Associated Adult T-Cell Leukemia. *J Virol* 89(23)**,** 11786-11800. doi: 10.1128/JVI.01356-15.

Bucheit, A.D., Kumar, S., Grote, D.M., Lin, Y., von Messling, V., Cattaneo, R.B., et al. (2003). An oncolytic measles virus engineered to enter cells through the CD20 antigen. *Mol Ther* 7(1)**,** 62-72. doi: 10.1016/s1525-0016(02)00033-3.

Castleton, A., Dey, A., Beaton, B., Patel, B., Aucher, A., Davis, D.M., et al. (2014). Human mesenchymal stromal cells deliver systemic oncolytic measles virus to treat acute lymphoblastic leukemia in the presence of humoral immunity. *Blood* 123(9)**,** 1327-1335. doi: 10.1182/blood-2013-09-528851.

Cesaire, R., Oliere, S., Sharif-Askari, E., Loignon, M., Lezin, A., Olindo, S., et al. (2006). Oncolytic activity of vesicular stomatitis virus in primary adult T-cell leukemia. *Oncogene* 25(3)**,** 349-358. doi: 10.1038/sj.onc.1209055.

Chen, C.Y., Senac, J.S., Weaver, E.A., May, S.M., Jelinek, D.F., Greipp, P., et al. (2011). Species D adenoviruses as oncolytics against B-cell cancers. *Clin Cancer Res* 17(21)**,** 6712-6722. doi: 10.1158/1078-0432.CCR-11-0968.

Contag, C.H., Sikorski, R., Negrin, R.S., Schmidt, T., Fan, A.C., Bachireddy, P., et al. (2010). Definition of an enhanced immune cell therapy in mice that can target stem-like lymphoma cells. *Cancer Res* 70(23)**,** 9837-9845. doi: 10.1158/0008-5472.CAN-10-2650.

Dey, A., Zhang, Y., Castleton, A.Z., Bailey, K., Beaton, B., Patel, B., et al. (2016). The Role of Neutrophils in Measles Virus-mediated Oncolysis Differs Between B-cell Malignancies and Is Not Always Enhanced by GCSF. *Mol Ther* 24(1)**,** 184-192. doi: 10.1038/mt.2015.149.

Dingli, D., Peng, K.W., Harvey, M.E., Greipp, P.R., O'Connor, M.K., Cattaneo, R., et al. (2004). Image-guided radiovirotherapy for multiple myeloma using a recombinant measles virus expressing the thyroidal sodium iodide symporter. *Blood* 103(5)**,** 1641-1646. doi: 10.1182/blood-2003-07-2233.

Esfandyari, T., Tefferi, A., Szmidt, A., Alain, T., Zwolak, P., Lasho, T., et al. (2009). Transcription factors down-stream of Ras as molecular indicators for targeting malignancies with oncolytic herpes virus. *Mol Oncol* 3(5-6)**,** 464-468. doi: 10.1016/j.molonc.2009.07.002.

Geletneky, K., Hajda, J., Angelova, A.L., Leuchs, B., Capper, D., Bartsch, A.J., et al. (2017). Oncolytic H-1 Parvovirus Shows Safety and Signs of Immunogenic Activity in a First Phase I/IIa Glioblastoma Trial. *Mol Ther* 25(12)**,** 2620-2634. doi: 10.1016/j.ymthe.2017.08.016.

Grote, D., Russell, S.J., Cornu, T.I., Cattaneo, R., Vile, R., Poland, G.A., et al. (2001). Live attenuated measles virus induces regression of human lymphoma xenografts in immunodeficient mice. *Blood* 97(12)**,** 3746-3754. doi: 10.1182/blood.v97.12.3746.

Hall, K., Scott, K.J., Rose, A., Desborough, M., Harrington, K., Pandha, H., et al. (2012). Reovirus-mediated cytotoxicity and enhancement of innate immune responses against acute myeloid leukemia. *Biores Open Access* 1(1)**,** 3-15. doi: 10.1089/biores.2012.0205.

Hanauer, J.D.S., Rengstl, B., Kleinlutzum, D., Reul, J., Pfeiffer, A., Friedel, T., et al. (2018). CD30-targeted oncolytic viruses as novel therapeutic approach against classical Hodgkin lymphoma. *Oncotarget* 9(16)**,** 12971-12981. doi: 10.18632/oncotarget.24191.

Haralambieva, I., Iankov, I., Hasegawa, K., Harvey, M., Russell, S.J., and Peng, K.W. (2007). Engineering oncolytic measles virus to circumvent the intracellular innate immune response. *Mol Ther* 15(3)**,** 588-597. doi: 10.1038/sj.mt.6300076.

Heinze, S., Stadler, G.L., Zhang, Y., Engeland, C.E., Barth, T.F.E., Heidbuechel, J.P.W., et al. (2026). A measles virus encoding a CD19/CD3 bispecific T cell engager shows enhanced preclinical anti-BCP-ALL efficacy without significant toxicity. *Mol Ther Oncol* 34(1)**,** 201127. doi: 10.1016/j.omton.2026.201127.

Heinzerling, L., Kunzi, V., Oberholzer, P.A., Kundig, T., Naim, H., and Dummer, R. (2005). Oncolytic measles virus in cutaneous T-cell lymphomas mounts antitumor immune responses in vivo and targets interferon-resistant tumor cells. *Blood* 106(7)**,** 2287-2294. doi: 10.1182/blood-2004-11-4558.

Holmes, M., Scott, G.B., Heaton, S., Barr, T., Askar, B., Muller, L.M.E., et al. (2023). Efficacy of coxsackievirus A21 against drug-resistant neoplastic B cells. *Mol Ther Oncolytics* 29**,** 17-29. doi: 10.1016/j.omto.2023.03.002.

Hwang, C.C., Umeki, S., Igase, M., Coffey, M., Noguchi, S., Okuda, M., et al. (2016). The effects of oncolytic reovirus in canine lymphoma cell lines. *Vet Comp Oncol* 14 Suppl 1**,** 61-73. doi: 10.1111/vco.12124.

Iankov, I.D., Blechacz, B., Liu, C., Schmeckpeper, J.D., Tarara, J.E., Federspiel, M.J., et al. (2007). Infected cell carriers: a new strategy for systemic delivery of oncolytic measles viruses in cancer virotherapy. *Mol Ther* 15(1)**,** 114-122. doi: 10.1038/sj.mt.6300020.

Ishino, R., Kawase, Y., Kitawaki, T., Sugimoto, N., Oku, M., Uchida, S., et al. (2021). Oncolytic Virus Therapy with HSV-1 for Hematological Malignancies. *Mol Ther* 29(2)**,** 762-774. doi: 10.1016/j.ymthe.2020.09.041.

Islam, S., Espitia, C.M., Persky, D.O., Carew, J.S., and Nawrocki, S.T. (2021). Targeting JAK/STAT Signaling Antagonizes Resistance to Oncolytic Reovirus Therapy Driven by Prior Infection with HTLV-1 in Models of T-Cell Lymphoma. *Viruses* 13(7). doi: 10.3390/v13071406.

Ji, L., Huang, J., Kang, Y., Wu, C., Chen, Y., Wu, Y., et al. (2025). Sophora flavescens-derived extracellular vesicles loaded with oncolytic vaccinia virus/IR1061 for NIR-II photoacoustic imaging guided multimodal treatment of diffuse large B-cell lymphoma. *Mater Today Bio* 34**,** 102177. doi: 10.1016/j.mtbio.2025.102177.

Kelly, K.R., Espitia, C.M., Mahalingam, D., Oyajobi, B.O., Coffey, M., Giles, F.J., et al. (2012). Reovirus therapy stimulates endoplasmic reticular stress, NOXA induction, and augments bortezomib-mediated apoptosis in multiple myeloma. *Oncogene* 31(25)**,** 3023-3038. doi: 10.1038/onc.2011.478.

Kelly, K.R., Espitia, C.M., Zhao, W., Wendlandt, E., Tricot, G., Zhan, F., et al. (2015). Junctional adhesion molecule-A is overexpressed in advanced multiple myeloma and determines response to oncolytic reovirus. *Oncotarget* 6(38)**,** 41275-41289. doi: 10.18632/oncotarget.5753.

Kennedy, B.E., Giacomantonio, M., Murphy, J.P., Cutler, S., Sadek, M., Konda, P., et al. (2022). NAD+ depletion enhances reovirus-induced oncolysis in multiple myeloma. *Mol Ther Oncolytics* 24**,** 695-706. doi: 10.1016/j.omto.2022.02.017.

Kim, M., Rahman, M.M., Cogle, C.R., and McFadden, G. (2015). Prevention of EBV lymphoma development by oncolytic myxoma virus in a murine xenograft model of post-transplant lymphoproliferative disease. *Biochem Biophys Res Commun* 462(4)**,** 283-287. doi: 10.1016/j.bbrc.2015.03.146.

Kunzi, V., Oberholzer, P.A., Heinzerling, L., Dummer, R., and Naim, H.Y. (2006). Recombinant measles virus induces cytolysis of cutaneous T-cell lymphoma in vitro and in vivo. *J Invest Dermatol* 126(11)**,** 2525-2532. doi: 10.1038/sj.jid.5700529.

Lei, W., Wang, S., Yang, C., Huang, X., Chen, Z., He, W., et al. (2016). Combined expression of miR-34a and Smac mediated by oncolytic vaccinia virus synergistically promote anti-tumor effects in Multiple Myeloma. *Sci Rep* 6**,** 32174. doi: 10.1038/srep32174.

Lei, W., Ye, Q., Hao, Y., Chen, J., Huang, Y., Yang, L., et al. (2022). CD19-targeted BiTE expression by an oncolytic vaccinia virus significantly augments therapeutic efficacy against B-cell lymphoma. *Blood Cancer J* 12(2)**,** 35. doi: 10.1038/s41408-022-00634-4.

Leveille, S., Goulet, M.L., Lichty, B.D., and Hiscott, J. (2011a). Vesicular stomatitis virus oncolytic treatment interferes with tumor-associated dendritic cell functions and abrogates tumor antigen presentation. *J Virol* 85(23)**,** 12160-12169. doi: 10.1128/JVI.05703-11.

Leveille, S., Samuel, S., Goulet, M.L., and Hiscott, J. (2011b). Enhancing VSV oncolytic activity with an improved cytosine deaminase suicide gene strategy. *Cancer Gene Ther* 18(6)**,** 435-443. doi: 10.1038/cgt.2011.14.

Li, M., Zhang, Y., Zong, L., Zhang, M., Wang, S., Lei, W., et al. (2025). An oncolytic vaccinia virus expressing anti-CD47 nanobody exerts enhanced antitumor activity by mediating innate and adaptive immune cell infiltration and activation in the lymphoma tumor microenvironment. *Haematologica* 110(9)**,** 2113-2128. doi: 10.3324/haematol.2024.286923.

Lou, J., Dong, J., Xu, R., Zeng, H., Fang, L., Wu, Y., et al. (2021). Remodeling of the tumor microenvironment using an engineered oncolytic vaccinia virus improves PD-L1 inhibition outcomes. *Biosci Rep* 41(6). doi: 10.1042/BSR20204186.

Luhl, N.C., Zirngibl, F., Dorneburg, C., Wei, J., Dahlhaus, M., Barth, T.F., et al. (2014). Attenuated measles virus controls pediatric acute B-lineage lymphoblastic leukemia in NOD/SCID mice. *Haematologica* 99(6)**,** 1050-1061. doi: 10.3324/haematol.2013.087205.

Marchica, V., Franceschi, V., Vescovini, R., Storti, P., Vicario, E., Toscani, D., et al. (2020). Bovine pestivirus is a new alternative virus for multiple myeloma oncolytic virotherapy. *J Hematol Oncol* 13(1)**,** 89. doi: 10.1186/s13045-020-00919-w.

MC, M.P., Fernandez, S.A., Landes, K., Huey, D., Lairmore, M., and Niewiesk, S. (2014). Success of measles virotherapy in ATL depends on type I interferon secretion and responsiveness. *Virus Res* 189**,** 206-213. doi: 10.1016/j.virusres.2014.05.025.

Miest, T.S., Frenzke, M., and Cattaneo, R. (2013). Measles virus entry through the signaling lymphocyte activation molecule governs efficacy of mantle cell lymphoma radiovirotherapy. *Mol Ther* 21(11)**,** 2019-2031. doi: 10.1038/mt.2013.171.

Mohammed, M.S., Al-Taee, M.F., and Al-Shammari, A.M. (2019). Caspase Dependent and Independent Anti-hematological Malignancy Activity of AMHA1 Attenuated Newcastle Disease Virus. *Int J Mol Cell Med* 8(3)**,** 211-223. doi: 10.22088/IJMCM.BUMS.8.3.211.

Muller, L.M.E., Migneco, G., Scott, G.B., Down, J., King, S., Askar, B., et al. (2021). Reovirus-induced cell-mediated immunity for the treatment of multiple myeloma within the resistant bone marrow niche. *J Immunother Cancer* 9(3). doi: 10.1136/jitc-2020-001803.

Naik, S., Galyon, G.D., Jenks, N.J., Steele, M.B., Miller, A.C., Allstadt, S.D., et al. (2018). Comparative Oncology Evaluation of Intravenous Recombinant Oncolytic Vesicular Stomatitis Virus Therapy in Spontaneous Canine Cancer. *Mol Cancer Ther* 17(1)**,** 316-326. doi: 10.1158/1535-7163.MCT-17-0432.

Naik, S., Nace, R., Federspiel, M.J., Barber, G.N., Peng, K.W., and Russell, S.J. (2012). Curative one-shot systemic virotherapy in murine myeloma. *Leukemia* 26(8)**,** 1870-1878. doi: 10.1038/leu.2012.70.

Ong, H.T., Timm, M.M., Greipp, P.R., Witzig, T.E., Dispenzieri, A., Russell, S.J., et al. (2006). Oncolytic measles virus targets high CD46 expression on multiple myeloma cells. *Exp Hematol* 34(6)**,** 713-720. doi: 10.1016/j.exphem.2006.03.002.

Parrish, C., Scott, G.B., Migneco, G., Scott, K.J., Steele, L.P., Ilett, E., et al. (2015). Oncolytic reovirus enhances rituximab-mediated antibody-dependent cellular cytotoxicity against chronic lymphocytic leukaemia. *Leukemia* 29(9)**,** 1799-1810. doi: 10.1038/leu.2015.88.

Parrula, C., Fernandez, S.A., Zimmerman, B., Lairmore, M., and Niewiesk, S. (2011). Measles virotherapy in a mouse model of adult T-cell leukaemia/lymphoma. *J Gen Virol* 92(Pt 6)**,** 1458-1466. doi: 10.1099/vir.0.028910-0.

Patel, B., Dey, A., Ghorani, E., Kumar, S., Malam, Y., Rai, L., et al. (2011). Differential cytopathology and kinetics of measles oncolysis in two primary B-cell malignancies provides mechanistic insights. *Mol Ther* 19(6)**,** 1034-1040. doi: 10.1038/mt.2011.44.

Peng, K.W., Donovan, K.A., Schneider, U., Cattaneo, R., Lust, J.A., and Russell, S.J. (2003). Oncolytic measles viruses displaying a single-chain antibody against CD38, a myeloma cell marker. *Blood* 101(7)**,** 2557-2562. doi: 10.1182/blood-2002-07-2195.

Porosnicu, M., Mian, A., and Barber, G.N. (2003). The oncolytic effect of recombinant vesicular stomatitis virus is enhanced by expression of the fusion cytosine deaminase/uracil phosphoribosyltransferase suicide gene. *Cancer Res* 63(23)**,** 8366-8376.

Qian, W., Liu, J., Tong, Y., Yan, S., Yang, C., Yang, M., et al. (2008). Enhanced antitumor activity by a selective conditionally replicating adenovirus combining with MDA-7/interleukin-24 for B-lymphoblastic leukemia via induction of apoptosis. *Leukemia* 22(2)**,** 361-369. doi: 10.1038/sj.leu.2405034.

Raykov, Z., Savelyeva, L., Balboni, G., Giese, T., Rommelaere, J., and Giese, N.A. (2005). B1 lymphocytes and myeloid dendritic cells in lymphoid organs are preferential extratumoral sites of parvovirus minute virus of mice prototype strain expression. *J Virol* 79(6)**,** 3517-3524. doi: 10.1128/JVI.79.6.3517-3524.2005.

Samuel, S., Beljanski, V., Van Grevenynghe, J., Richards, S., Ben Yebdri, F., He, Z., et al. (2013). BCL-2 inhibitors sensitize therapy-resistant chronic lymphocytic leukemia cells to VSV oncolysis. *Mol Ther* 21(7)**,** 1413-1423. doi: 10.1038/mt.2013.91.

Sanchez, D., Pelayo, R., Medina, L.A., Vadillo, E., Sanchez, R., Nunez, L., et al. (2015). Newcastle Disease Virus: Potential Therapeutic Application for Human and Canine Lymphoma. *Viruses* 8(1). doi: 10.3390/v8010003.

Schache, P., Gurlevik, E., Struver, N., Woller, N., Malek, N., Zender, L., et al. (2009). VSV virotherapy improves chemotherapy by triggering apoptosis due to proteasomal degradation of Mcl-1. *Gene Ther* 16(7)**,** 849-861. doi: 10.1038/gt.2009.39.

Shen, W., Patnaik, M.M., Ruiz, A., Russell, S.J., and Peng, K.W. (2016). Immunovirotherapy with vesicular stomatitis virus and PD-L1 blockade enhances therapeutic outcome in murine acute myeloid leukemia. *Blood* 127(11)**,** 1449-1458. doi: 10.1182/blood-2015-06-652503.

Stiff, A., Caserta, E., Sborov, D.W., Nuovo, G.J., Mo, X., Schlotter, S.Y., et al. (2016). Histone Deacetylase Inhibitors Enhance the Therapeutic Potential of Reovirus in Multiple Myeloma. *Mol Cancer Ther* 15(5)**,** 830-841. doi: 10.1158/1535-7163.MCT-15-0240-T.

Suter, S.E., Chein, M.B., von Messling, V., Yip, B., Cattaneo, R., Vernau, W., et al. (2005). In vitro canine distemper virus infection of canine lymphoid cells: a prelude to oncolytic therapy for lymphoma. *Clin Cancer Res* 11(4)**,** 1579-1587. doi: 10.1158/1078-0432.CCR-04-1944.

Suzme, R., Tseng, J.C., Levin, B., Ibrahim, S., Meruelo, D., and Pellicer, A. (2012). Sindbis viral vectors target hematopoietic malignant cells. *Cancer Gene Ther* 19(11)**,** 757-766. doi: 10.1038/cgt.2012.56.

Takeda, S., Kanbayashi, D., Kurata, T., Yoshiyama, H., and Komano, J. (2014). Enhanced susceptibility of B lymphoma cells to measles virus by Epstein-Barr virus type III latency that upregulates CD150/signaling lymphocytic activation molecule. *Cancer Sci* 105(2)**,** 211-218. doi: 10.1111/cas.12324.

Thirukkumaran, C.M., Luider, J.M., Stewart, D.A., Cheng, T., Lupichuk, S.M., Nodwell, M.J., et al. (2003). Reovirus oncolysis as a novel purging strategy for autologous stem cell transplantation. *Blood* 102(1)**,** 377-387. doi: 10.1182/blood-2002-08-2508.

Thirukkumaran, C.M., Shi, Z.Q., Luider, J., Kopciuk, K., Bahlis, N., Neri, P., et al. (2014). Reovirus as a successful ex vivo purging modality for multiple myeloma. *Bone Marrow Transplant* 49(1)**,** 80-86. doi: 10.1038/bmt.2013.130.

Thirukkumaran, C.M., Shi, Z.Q., Luider, J., Kopciuk, K., Gao, H., Bahlis, N., et al. (2012). Reovirus as a viable therapeutic option for the treatment of multiple myeloma. *Clin Cancer Res* 18(18)**,** 4962-4972. doi: 10.1158/1078-0432.CCR-11-3085.

Thirukkumaran, C.M., Shi, Z.Q., Luider, J., Kopciuk, K., Gao, H., Bahlis, N., et al. (2013). Reovirus modulates autophagy during oncolysis of multiple myeloma. *Autophagy* 9(3)**,** 413-414. doi: 10.4161/auto.22867.

Tumilasci, V.F., Oliere, S., Nguyen, T.L., Shamy, A., Bell, J., and Hiscott, J. (2008). Targeting the apoptotic pathway with BCL-2 inhibitors sensitizes primary chronic lymphocytic leukemia cells to vesicular stomatitis virus-induced oncolysis. *J Virol* 82(17)**,** 8487-8499. doi: 10.1128/JVI.00851-08.

Tzadok-David, Y., Metzkin-Eizenberg, M., and Zakay-Rones, Z. (1995). The effect of a mesogenic and a lentogenic Newcastle disease virus strain on Burkitt lymphoma Daudi cells. *J Cancer Res Clin Oncol* 121(3)**,** 169-174. doi: 10.1007/BF01198099.

Ungerechts, G., Frenzke, M.E., Yaiw, K.C., Miest, T., Johnston, P.B., and Cattaneo, R. (2010). Mantle cell lymphoma salvage regimen: synergy between a reprogrammed oncolytic virus and two chemotherapeutics. *Gene Ther* 17(12)**,** 1506-1516. doi: 10.1038/gt.2010.103.

Ungerechts, G., Springfeld, C., Frenzke, M.E., Lampe, J., Johnston, P.B., Parker, W.B., et al. (2007). Lymphoma chemovirotherapy: CD20-targeted and convertase-armed measles virus can synergize with fludarabine. *Cancer Res* 67(22)**,** 10939-10947. doi: 10.1158/0008-5472.CAN-07-1252.

Wang, G., Li, G., Liu, H., Yang, C., Yang, X., Jin, J., et al. (2009). E1B 55-kDa deleted, Ad5/F35 fiber chimeric adenovirus, a potential oncolytic agent for B-lymphocytic malignancies. *J Gene Med* 11(6)**,** 477-485. doi: 10.1002/jgm.1326.

Wang, P., Wu, Y., Yang, C., Zhao, G., Liu, Y., Cheng, G., et al. (2020). Embelin Promotes Oncolytic Vaccinia Virus-Mediated Antitumor Immunity Through Disruption of IL-6/STAT3 Signaling in Lymphoma. *Onco Targets Ther* 13**,** 1421-1429. doi: 10.2147/OTT.S209312.

Weaver, E.A., Chen, C.Y., May, S.M., Barry, M.E., and Barry, M.A. (2011). Comparison of adenoviruses as oncolytics and cancer vaccines in an immunocompetent B cell lymphoma model. *Hum Gene Ther* 22(9)**,** 1095-1100. doi: 10.1089/hum.2011.071.

Wenthe, J., Naseri, S., Hellstrom, A.C., Wiklund, H.J., Eriksson, E., and Loskog, A. (2020). Immunostimulatory oncolytic virotherapy for multiple myeloma targeting 4-1BB and/or CD40. *Cancer Gene Ther* 27(12)**,** 948-959. doi: 10.1038/s41417-020-0176-9.

Wenthe, J., Naseri, S., Labani-Motlagh, A., Enblad, G., Wikstrom, K.I., Eriksson, E., et al. (2021). Boosting CAR T-cell responses in lymphoma by simultaneous targeting of CD40/4-1BB using oncolytic viral gene therapy. *Cancer Immunol Immunother* 70(10)**,** 2851-2865. doi: 10.1007/s00262-021-02895-7.

Wu, H., Zhang, J., Xu, L., Xie, Y., Pan, L., Zhang, X., et al. (2026). IL-18-armed oncolytic vaccinia virus remodels the suppressive microenvironment via macrophage and Treg modulation in lymphoma. *Cancer Immunol Immunother* 75(5). doi: 10.1007/s00262-026-04396-x.

Xie, S., Fan, W., Yang, C., Lei, W., Pan, H., Tong, X., et al. (2021). Beclin1‑armed oncolytic Vaccinia virus enhances the therapeutic efficacy of R‑CHOP against lymphoma in vitro and in vivo. *Oncol Rep* 45(3)**,** 987-996. doi: 10.3892/or.2021.7942.

Yaiw, K.C., Miest, T.S., Frenzke, M., Timm, M., Johnston, P.B., and Cattaneo, R. (2011). CD20-targeted measles virus shows high oncolytic specificity in clinical samples from lymphoma patients independent of prior rituximab therapy. *Gene Ther* 18(3)**,** 313-317. doi: 10.1038/gt.2010.150.

Yang, A., Wang, X., Jin, L., Luo, H., Yang, Z., Yang, N., et al. (2024). Human umbilical cord mesenchymal stem cell exosomes deliver potent oncolytic reovirus to acute myeloid leukemia cells. *Virology* 598**,** 110171. doi: 10.1016/j.virol.2024.110171.

Yu, M., Scherwitzl, I., Opp, S., Tsirigos, A., and Meruelo, D. (2019). Molecular and metabolic pathways mediating curative treatment of a non-Hodgkin B cell lymphoma by Sindbis viral vectors and anti-4-1BB monoclonal antibody. *J Immunother Cancer* 7(1)**,** 185. doi: 10.1186/s40425-019-0664-3.

Zhang, B., Shu, Y., Hu, S., Qi, Z., Chen, Y., Ma, J., et al. (2022). In Situ Tumor Vaccine Expressing Anti-CD47 Antibody Enhances Antitumor Immunity. *Front Oncol* 12**,** 897561. doi: 10.3389/fonc.2022.897561.

Zhang, J., Guo, Y., Fang, H., Guo, X., and Zhao, L. (2023). Oncolytic virus oHSV2 combined with PD-1/PD-L1 inhibitors exert antitumor activity by mediating CD4 + T and CD8 + T cell infiltration in the lymphoma tumor microenvironment. *Autoimmunity* 56(1)**,** 2259126. doi: 10.1080/08916934.2023.2259126.

Zhang, X., Zhao, L., Hang, Z., Guo, H., and Zhang, M. (2011). Evaluation of HSV-1 and adenovirus vector-mediated infection, replication and cytotoxicity in lymphoma cell lines. *Oncol Rep* 26(3)**,** 637-644. doi: 10.3892/or.2011.1306.
