# Supplementary material for: Therapeutic potential of oncolytic viruses in hematopoietic malignancies: from preclinical insights to clinical realities
Source: Front Microbiol. 2026 Sep 29;17:1925346. doi: 10.3389/fmicb.2026.1925346 (PMC13659887; doi:10.3389/fmicb.2026.1925346)
Supplement: Supplementary file 2 [file Table_2.docx]

**Supplementary table 2. Clinical trials and case reports evaluating oncolytic viral therapy to treat hematopoietic malignant.**

| Ovs (Name, virus type, construct) | Conditions | Therapy Strategy / aims | Achievement /outcome | Phase  Trial duration  ClinicalTrials ID |
| --- | --- | --- | --- | --- |
| **Normal clinical trial** | | | | |
| Reolysin (reovirus) | Relapsed or refractory multiple myeloma | Determine safety and tolerability;  Obtain preliminary data on response; | no dose-limiting toxicities;  Viral RNA confined to myeloma cells;  monotherapy insufficient | Phase I  2012-2015  NCT01533194  (Sborov et al., 2014) |
| MV-NIS  (thyroidal sodium iodide symporter) | Relapsed/refractory multiple myeloma | To determine the maximum tolerated dose (MTD) of MV-NIS with/out cyclophosphamide.  To determine viral gene expression, the biodistribution of virally infected cells at various times points after infection | MV-NIS is capable of replicating before being cleared by the immune system;  systemic administration in patients with myeloma as a safe and novel approach;  MTD not reached;  One case of complete response and disease free period up to 9 months (after irradiation). | Phase I  2006 -2019  NCT00450814  (Dispenzieri et al., 2017) |
| MV-NIS | Relapsed/ refractory multiple myeloma | One dose of the OV in conjunction with a 4-day course cyclophosphamide application intravenously To assess the effectiveness of the OV therapy in combination with cyclophosphamide for patients with MM | NA | Phase II 2015-2019 NCT02192775 |
| Pelareorep  (Reovirus) | Relapsed multiple myeloma | Study of best dose and side effects of Pelareorep when administered (if.v.) with antineoplastic agents:  (carfilzomib + nivolumab + dexamethasone); A 3 Arm study: ARM I only anti neoplastic drugs administered; ARM II & III Pelareorep added | NA | Phase I  2018 - 2022 NCT03605719 |
| Pelareorep (Reovirus) | Relapsed/ refractory multiple myeloma | 13 heavily pretreated with MM. Patients received intravenous dexamethasone, carfilzomib, and Pelareorep intravenously | Bone marrow biopsies from 9 patients confirmed active Reovirus replication inside the MM cells, which was not seen in previous trials using Reovirus alone. The treatment also induced classical monocytic expansion, CD8+ T cell activation, caspase-3 activation indicating cell death and increased PD-L1 expression. | Phase Ib 2014-2021 NCT02101944 (Dona et al., 2025) |
| Pelareorep (Reovirus) | Relapsed/ refractory multiple myeloma | 14 patients with MM received dexamethasone, bortezomib and Pelareorep intravenously | 6 patients showed decreased paraprotein. The treatment increased T and natural killer cell activation, inflammatory cytokine release, and PD-L1expression in bone marrow | Phase I  2015-2022 NCT02514382 (Nawrocki et al., 2023) |
| T-VEC | Primary cutaneous B-cell lymphoma | To investigate T-VEC-induced local and systemic immune changes and its treatment efficacy. Median of 7 OV injections intratumoral. Pre/ post analysis of skin lesions through fine needle aspirate.  Patients treated with R-CHOP or rituximab monotherapy, before the trial. | 11 out of 13 patients showed positive tumor response. Complete clinical response in 6 patients. 5 others showed partial response and two showed disease progression;  None developed herpes simplex-associated infection. In all responsive FNA of injected lesions, a decline of B-cells can be observed;  By the end of the therapy the decline of B cells was observed in non-injected lesions as well; Additionally, the number of NK-cells, monocytes and T-cells increased in the lesions, indicating a targeted immune response. | Phase I  2018-2022  NCT03458117 (Ramelyte et al., 2021) |
| VSV-hIFNβ-NIS | Relapsed Refractory MM, TCL, AML | To determine maximum tolerated dose and estimate clinical response rate;  IV administered; VSV-hIFNβ-NIS vaccine with or without cyclophosphamide and combinations of ipilumab/nivolumab/cemiplimab. | Tolerated with no dose limiting toxicities;  5 out of 7 patients with TCL showed at least objective result, one with complete response; Efficacy appeared dose-dependent. Most responsive at highest does level (DL4); | Phase I  2017 - est. 2032  NCT03017820 (Cook et al., 2022) |
| RT-01  vaccinia virus (VV) | Relapsed or refractory T-cell lymphoma | RT-01 is administered intravenously on day 1 and 6, and every 8 weeks thereafter (up to 6 times) | NA | Phase I  2022 -pending  NCT05387226 |
| RGV004  VV | B-Cell Lymphoma | aims to evaluate the maximum tolerated dose (MTD) and dose-dependent toxicity (DLT); (3×3 design) | NA | Phase I  2022 - est. 2025 NCT04887025 |
| RGV005 VV | Refractory/ relapsed  B-Cell lymphoma | single-dose escalation safety and tolerability trial (3×3 design); four dose groups and two delivery groups: -intratumoral injection group -intravenous injection group | NA | Phase I  2026 - est. 2029 NCT07398963 |
| **Case Report** | | | | |
| MV-NIS | Relapsing/ refractory myeloma and multiple glucose-avid plasmacytomas | 2 measles-seronegative patients received intravenous infusion of 10(11) TCID50 (50% tissue culture infectious dose) infectious units of MV-NIS | -Both patients responded to therapy with protein reduction and resolution of bone marrow plasmacytosis.  -one patient experienced durable complete remission at all disease sites | (Russell et al., 2014) |
| T-VEC | Adult T-cell leukemia/ lymphoma (ATLL) with human T-cell leukemia virus type 1 (HTLV-1) | Patient underwent multiple Therapies before. After partial response, the disease progressed. Single-cell RNA sequencing pre/post treatment through FNA of lesions; Injection of T-VEC and adjuvant Mogamulizumab / 4Gy Radiation | Reduction of lesion size and stabilization of patient’s condition Patients survival prolonged by 5 months, ultimately progressing T-VEC exerts oncolysis with activation of immune response in immunosuppressive tumor micro-environment | (An et al., 2025) |
| SARS-CoV-2 | Refractory NK/ T-cell lymphoma  associated with  EBV and AIHA | Contraction of Covid-19 during relapse of NK/ T-cell Lymphoma with prominent clinical parameters. Patient received several RBC transfusions, methylprednisolone, levofloxacin | On day 11 of onset, spontaneous steady clinical improvement: increase of hemoglobin level, plasma EBV-DNA dropped, reduction of splenomegaly, remarkable reduction of NK-cell population.  On day 34: after negative oropharyngeal swab for SARS-Cov-2 steady relapse of the primary condition with the parameters mentioned above. | (Pasin et al., 2020) |
| SARS-CoV-2 | High-grade B-cell lymphoma | Contraction of Covid-19 occurred when second R-CHOP cycle was due. Favipiravir administered after a positive PCR analysis. | Images of PET-CT on day 24 of infection compared to images upon Lymphoma diagnosis, reveal considerable disappearance of pathological lymph node and intestinal wall thickening.  Observation raises the possibility SARS-Cov-2 could have played a major role in declining lymphoma progression after just one R-CHOP cycle. | (Yilmaz et al., 2021) |
| SARS-CoV-2 | Acute myeloid leukemia T-cell Acute lymphoblastic leukemia | Contraction of Covid-19 delayed chemotherapy. Meanwhile patients only received supportive therapy (azithromycin/ fluconazole/ prednisolone) until remission from infection. | Routine diagnostic blood work revealed drop of blast cell counts from 30% to 3% in AML case and 0% in T-cell ALL case.  SARS-Cov-2 might possess oncolytic properties with tumor antigen release or convey immune response through cross reactivity with tumor antigens. | (Kandeel et al., 2021) |

An, J., Hu, E., Shi, Y., Fang, Y., Liu, N., Liu, Q., et al. (2025). Single-cell analysis of oncolytic virotherapy in adult T-cell leukemia/lymphoma. *J Immunother Cancer* 13(3). doi: 10.1136/jitc-2024-011265.

Cook, J., Peng, K.W., Witzig, T.E., Broski, S.M., Villasboas, J.C., Paludo, J., et al. (2022). Clinical activity of single-dose systemic oncolytic VSV virotherapy in patients with relapsed refractory T-cell lymphoma. *Blood Adv* 6(11)**,** 3268-3279. doi: 10.1182/bloodadvances.2021006631.

Dona, A.A., Tandoh, T., Nigam, L., Singer, M., Caserta, E., Murtadha, M., et al. (2025). Proteasome inhibition enhances oncolytic reovirus therapy in multiple myeloma independently of its direct cytotoxic effects. *Journal of Hematology & Oncology* 18 (1), doi: 10.1186/s13045-024-01645-3

Dispenzieri, A., Tong, C., LaPlant, B., Lacy, M.Q., Laumann, K., Dingli, D., et al. (2017). Phase I trial of systemic administration of Edmonston strain of measles virus genetically engineered to express the sodium iodide symporter in patients with recurrent or refractory multiple myeloma. *Leukemia* 31(12)**,** 2791-2798. doi: 10.1038/leu.2017.120.

Kandeel, E.Z., Refaat, L., Abdel-Fatah, R., Samra, M., Bayoumi, A., Abdellateif, M.S., et al. (2021). Could COVID-19 induce remission of acute leukemia? *Hematology* 26(1)**,** 870-873. doi: 10.1080/16078454.2021.1992117.

Nawrocki, S.T., Olea, J., Celi, C.V., Dadrastoussi, H., Wu, K., Tsao-Wei, D., et al. (2023). Comprehensive Single-Cell Immune Profiling Defines the Patient Multiple Myeloma Microenvironment Following Oncolytic Virus Therapy in a Phase Ib Trial *Clinical Cancer Research* 29 (24), 5087-5103. doi: 10.1158/1078-0432.CCR-23-0229

Pasin, F., Mascalchi Calveri, M., Calabrese, A., Pizzarelli, G., Bongiovanni, I., Andreoli, M., et al. (2020). Oncolytic effect of SARS-CoV2 in a patient with NK lymphoma. *Acta Biomed* 91(3), ahead of print. doi: 10.23750/abm.v91i3.10141.

Ramelyte, E., Tastanova, A., Balazs, Z., Ignatova, D., Turko, P., Menzel, U., et al. (2021). Oncolytic virotherapy-mediated anti-tumor response: a single-cell perspective. *Cancer Cell* 39(3), 394-406 e394. doi: 10.1016/j.ccell.2020.12.022.

Russell, S.J., Federspiel, M.J., Peng, K.W., Tong, C., Dingli, D., Morice, W.G., et al. (2014). Remission of disseminated cancer after systemic oncolytic virotherapy. *Mayo Clin Proc* 89(7)**,** 926-933. doi: 10.1016/j.mayocp.2014.04.003.

Sborov, D.W., Nuovo, G.J., Stiff, A., Mace, T., Lesinski, G.B., Benson, D.M., Jr., et al. (2014). A phase I trial of single-agent reolysin in patients with relapsed multiple myeloma. *Clin Cancer Res* 20(23)**,** 5946-5955. doi: 10.1158/1078-0432.CCR-14-1404.

Yilmaz, F., Yasar, S., Tuncali, M.C., and Akin, S. (2021). Complete response in a frail patient with high-grade B-cell lymphoma to only one cycle of R-CHOP or to prolonged COVID-19? *Semin Oncol* 48(4-6)**,** 279-282. doi: 10.1053/j.seminoncol.2021.11.001.
